# Orai1 Channel Inhibition Preserves Left Ventricular Systolic Function and Normal Ca^2+^ Handling After Pressure Overload

**DOI:** 10.1161/CIRCULATIONAHA.118.038891

**Published:** 2020-01-07

**Authors:** Fiona Bartoli, Marc A. Bailey, Baptiste Rode, Philippe Mateo, Fabrice Antigny, Kaveen Bedouet, Pascale Gerbaud, Rajendra Gosain, Jeffrey Plante, Katherine Norman, Susana Gomez, Florence Lefebvre, Catherine Rucker-Martin, Justin F.X. Ainscough, Mark T. Kearney, Alexander-Francisco Bruns, Jian Shi, Hollie L. Appleby, Richard S. Young, Heba M. Shawer, Marjolaine Debant, Ana-Maria Gomez, David J. Beech, Richard Foster, Jean-Pierre Benitah, Jessica Sabourin

**Affiliations:** 1 Inserm, UMR-S 1180, Signalisation et Physiopathologie Cardiovasculaire, Université Paris-Saclay, Châtenay-Malabry, France (F.B., P.M., K.B., P.G., S.G., F.L., A.-M.G., J.P.B., J. Sabourin).; 2 Leeds Institute of Cardiovascular and Metabolic Medicine, School of Medicine, University of Leeds, United Kingdom (M.A.B., B.R., J.F.X.A., M.T.K., A.-F.B., J. Shi, H.L.A., R.S.Y., H.M.S., M.D., D.J.B.).; 3 School of Chemistry, University of Leeds, United Kingdom (R.G., J.P., K.N., R.F.).; 4 Inserm, UMR-S 999, Université Paris-Saclay, Centre Chirurgical Marie Lannelongue, Le Plessis-Robinson, France (F.A., C.R.M.).

**Keywords:** calcium, cardiomyocytes, hypertrophy, ORAI1 protein, ventricular function

## Abstract

**Background:**

Orai1 is a critical ion channel subunit, best recognized as a mediator of store-operated Ca^2+^ entry (SOCE) in nonexcitable cells. SOCE has recently emerged as a key contributor of cardiac hypertrophy and heart failure but the relevance of Orai1 is still unclear.

**Methods:**

To test the role of these Orai1 channels in the cardiac pathophysiology, a transgenic mouse was generated with cardiomyocyte-specific expression of an ion pore-disruptive Orai1^R91W^ mutant (C-dnO1). Synthetic chemistry and channel screening strategies were used to develop 4-(2,5-dimethoxyphenyl)-N-[(pyridin-4-yl)methyl]aniline (hereafter referred to as JPIII), a small-molecule Orai1 channel inhibitor suitable for in vivo delivery.

**Results:**

Adult mice subjected to transverse aortic constriction (TAC) developed cardiac hypertrophy and reduced ventricular function associated with increased Orai1 expression and Orai1-dependent SOCE (assessed by Mn^2+^ influx). C-dnO1 mice displayed normal cardiac electromechanical function and cellular excitation-contraction coupling despite reduced Orai1-dependent SOCE. Five weeks after TAC, C-dnO1 mice were protected from systolic dysfunction (assessed by preserved left ventricular fractional shortening and ejection fraction) even if increased cardiac mass and prohypertrophic markers induction were observed. This is correlated with a protection from TAC-induced cellular Ca^2+^ signaling alterations (increased SOCE, decreased [Ca^2+^]_i_ transients amplitude and decay rate, lower SR Ca^2+^ load and depressed cellular contractility) and SERCA2a downregulation in ventricular cardiomyocytes from C-dnO1 mice, associated with blunted Pyk2 signaling. There was also less fibrosis in heart sections from C-dnO1 mice after TAC. Moreover, 3 weeks treatment with JPIII following 5 weeks of TAC confirmed the translational relevance of an Orai1 inhibition strategy during hypertrophic insult.

**Conclusions:**

The findings suggest a key role of cardiac Orai1 channels and the potential for Orai1 channel inhibitors as inotropic therapies for maintaining contractility reserve after hypertrophic stress.

## Abstract 2

Supplemental Digital Content is available in the text.

**Clinical Perspective**

**What Is New?**

- Whereas store-operated Ca^2+^ entry has recently gained attention in cardiac pathophysiology, the role of the prototypic store-operated channel, Orai1 remains elusive.
- Using a novel genetically-modified mouse that specifically disrupts the Orai1 channel in cardiomyocytes, we showed that even if Orai1 is not instrumental in regulating normal excitation-contraction coupling and cardiac function, its functional inhibition preserves alterations of Ca^2+^ homeostasis, fibrosis and systolic function without affecting hypertrophy during pressure overload.
- JPIII, a novel in vivo–suitable small-molecule Orai1 channel inhibitor, markedly improves left ventricular systolic function and Ca^2+^ handling after pressure overload without causing adverse effect.

**What Are the Clinical Implications?**

- Ca^2+^ effectors have a major impact on cardiac hypertrophic signaling.
- We provide an important breakthrough that inhibiting Orai1 function in the heart could be beneficial after insult by protecting it against progressive left ventricular systolic dysfunction.
- Our results suggest that Orai1 inhibition has a potential favorable hemodynamic value to protect the heart from maladaptive hypertrophy and might represent a new inotropic support to help to relieve systolic dysfunction.

Despite significant management and therapeutic advances, heart failure (HF) still represents an epidemic threat, highlighting the need to deeper understand the cellular mechanisms involved in the pathogenesis in order to identify new molecular targets and develop innovative therapeutic strategies.

Recent studies reported that store-operated Ca^2+^ entry (SOCE) dysfunction contributes to the development of pathological cardiac hypertrophy and HF.^1,2^

The Orai1 protein (calcium release-activated calcium channel protein 1) has been proposed as the prototype of the pore-forming subunit of the plasmalemmal store-operated Ca^2+^ channels.^3^ While in nonexcitable cells Orai1/STIM1 (stromal interaction molecule 1)-mediated SOCE and its long-term pathophysiological outcomes have been relatively easier to pinpoint, its role in the heart is less clear. Orai1 and STIM1 have been detected in rodents and human heart.^4–6^ Orai1/STIM1-mediated SOCE appears to be developmentally regulated in the heart, with prominent activity in developing cardiomyocytes^7^ and plays a role in maintaining ventricular diastolic Ca^2+^ homeostasis that is crucial for postnatal cardiac growth.^4,8,9^ However, while Orai1-deficient zebrafish developed spontaneous HF,^5^ global heterozygous Orai1^+/-^ mice showed normal systolic function at baseline but developed more severe dilated cardiomyopathy after pressure overload insult.^10^

On the other hand, in vitro data showed Orai1 upregulation after hypertrophic stress and demonstrated that Orai1 silencing is associated with protection from hypertrophy via reduced calcineurin (CaN) activity.^4,11^ Likewise, it has been recently reported that in vivo overexpression of SOCE-associated regulatory factor in the heart prevents cardiac hypertrophy probably through suppressing the upregulation of STIM1 and Orai1.^12^

Consequently, the question remains whether this deleterious effect is related to developmental or adult systemic or cardiac specific alteration. Overall, the contribution of Orai1 to Ca^2+^ homeostasis and cardiac function remains elusive and conflicting.

The goal of the present study was to use novel tools to determine whether the cardiac Orai1-dependent pathway contributes to the electromechanical phenotype of ventricular cardiomyocytes using 2 independent approaches: (1) a genetic mouse model with cardiomyocyte-specific expression of the dominant-negative dn-Orai1^R91W^ mutant (C-dnO1) resulting in abolished store-operated Ca^2+^ channel function; and (2) pharmacological blockade by a novel Orai1 inhibitor, 4-(2,5-dimethoxyphenyl)-N-[(pyridin-4-yl)methyl]aniline (hereafter referred to as JPIII), which is suitable for in vivo use.

Our findings support the idea that Orai1 inhibition can improve Ca^2+^ cycling and systolic cardiac function during hypertrophic stress, suggesting that selective Orai1 blockade might provide a new therapy for cardiac hypertrophy and HF.

## Methods

All data and materials supporting the findings of this study are available in the article and its online-only Data Supplement or from the corresponding authors upon reasonable request.

All experiments were performed according to the ethical principles set by the French Ministry of Agriculture (agreement D-92–019-01) and the UK Scientific Procedures Act (1986) and were performed in accordance to the guidelines from Directive 2010/63/EU of the European Parliament on the protection of animals. Animal studies were approved by the local Ethics Committee (CREEA Ile-de-France Sud or Leeds Animal Welfare and Research Ethics Board and UK Home Office [PPL 40/3557 and P606320FB]).

Detailed descriptions of all materials and methods used in this study, including chemicals, generation of C-dnO1 transgenic mice, ventricular myocytes isolation, cell culture, Orai1 knockdown in pulmonary arterial smooth muscle cells, generation of Orai1 deficient fibroblasts, blue-gal staining, quantitative reverse transcription polymerase chain reaction with specific primers (Table I in the online-only Data Supplement), western blot with used antibodies (Table II in the online-only Data Supplement), immunostaining, immunohistochemistry of heart sections, histological analysis of cardiac fibrosis, measurement of cation influx using Fura-2 fluorescence quenching by MnCl_2_, Ca^2+^ imaging, high throughput Ca^2+^ imaging, action potential recording, patch-clamp recording, telemetric electrocardiogram studies, transverse aortic constriction, echocardiography, tail cuff plethysmography, liquid chromatography–mass spectrometry for JPIII, in vitro ADMET, in vivo chronic treatment, chemical synthesis are presented in online-only Data Supplement.

### Statistical Analysis

The number of cells (n) or animal (N) studied per experiment is indicated. Data are presented as mean±SEM. Statistical analyses were performed with Student *t* test, 1-way or 2-way ANOVA followed by post hoc Fisher LSD test for multiple comparisons, as stated in figures and tables legends, using GraphPad Prism 6.0 software. The equality of variance assumption was tested using an F-test and Brown-Forsythe test for the *t* test and ANOVA, respectively. All the assumptions of the equal variances were met. A value of *P*<0.05 was considered significant.

## Results

### Increased Orai1 Channel Expression and Activity After Transverse Aortic Constriction

**Figure 1.**
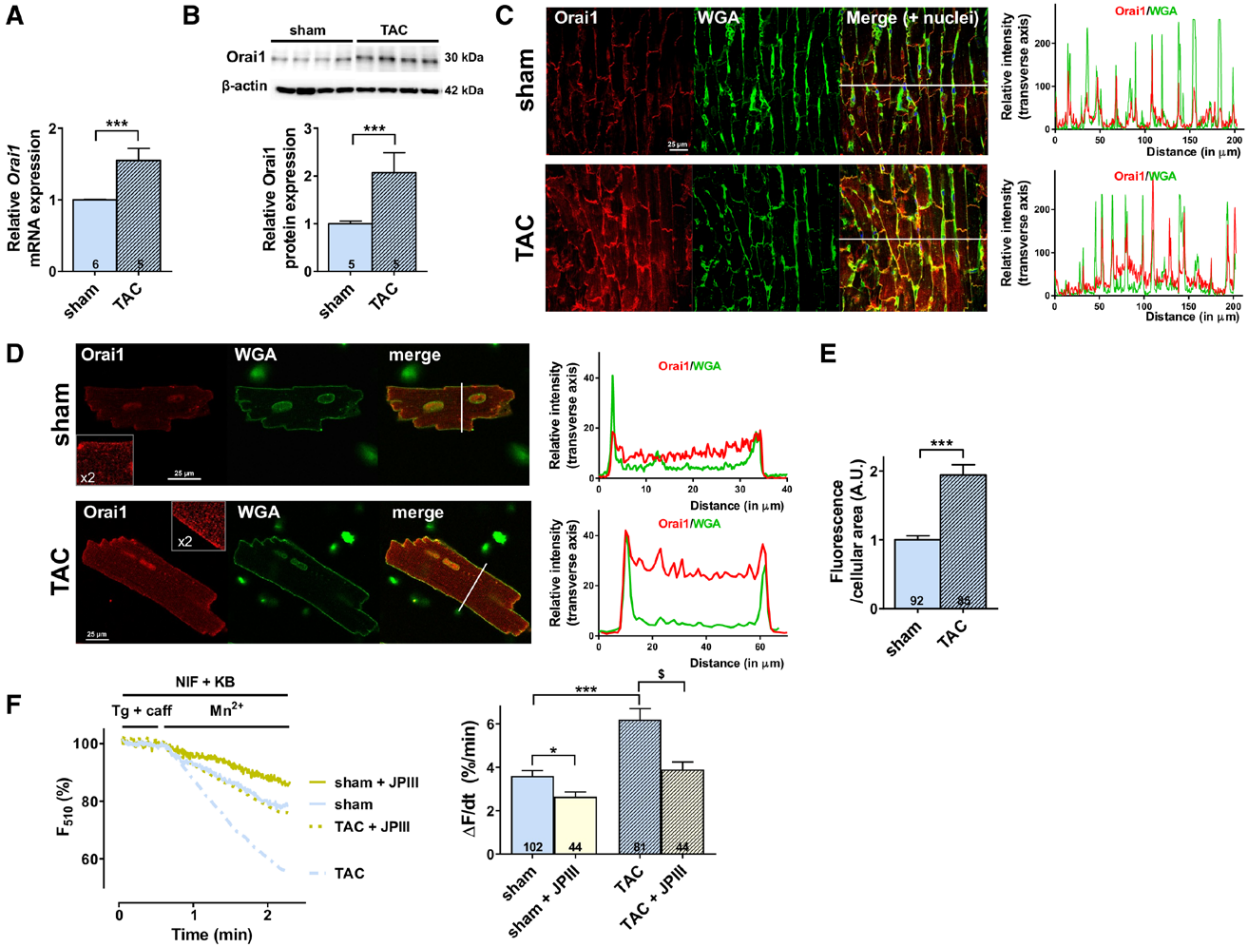
**TAC induces increased Orai1 expression and enhanced store-operated cation entry after 5 weeks**. **A**, Orai1 mRNA expression was determined by real-time quantitative polymerase chain reaction in ventricular tissue from sham-operated or transverse aortic constriction (TAC)-operated mice after 5 weeks. mRNA levels were normalized to housekeeping genes and expressed as fold change of that determined in sham mice. N=5–6 animals. ***P<0.001 vs sham mice. **B**, Top panel: representative western-blot of Orai1 in ventricular tissue from sham and TAC mice. Bottom panel: relative Orai1 protein expression was evaluated by western-blot. Protein levels were normalized by β-actin and expressed as fold change of that determined in sham mice. N=5 animals. ***P<0.001 vs sham mice. **C**, Left panel: representative immunohistochemistry with antibodies against Orai1 (red), WGA (wheat germ agglutinin; green) and nuclei (blue) showing Orai1 expression in mouse ventricular sections in sham and TAC conditions. Scale bar, 25 μm. Right panel: profile analysis from the whole transverse image demonstrates colocalization with WGA in peripheral regions. **D**, Representative immunostaining of Orai1 (red) and WGA (green) in ventricular cardiomyocytes from sham (top panel) or TAC (bottom panel) mice. Insets represent 2-fold magnification of main images. Profile analysis demonstrates colocalization with WGA in peripheral regions. White lines across ventricular cells (in transverse axis) show examples of regions from which pixel counts were taken. Scale bar, 25 μm. **E**, Quantification of Orai1 immunostaining in ventricular cells from sham and TAC mice. N=3 animals. n=85–92 investigated cells. ***P<0.001 vs sham mice. **F**, Left panel: representative fluorescence traces of the Fura-2 decay phase upon Mn^2+^ addition in cardiomyocytes from sham (light blue trace) and TAC (dotted light blue trace) mice in presence or absence of 5 µM JPIII, a new selective Orai1 inhibitor: sham (yellow trace) and after TAC (dotted yellow trace). Right panel: the initial slope of the Mn^2+^-induced decrease of Fura-2 fluorescence was fitted by linear regression and averaged (ΔF/dt, %/min) were presented on bar graph for cardiomyocytes from sham and TAC mice in presence or absence of 5 µM JPIII. N=4–6 animals. n=44–102 investigated cells. *P<0.05, ***P<0.001 vs sham cardiomyocytes. ^$^*P*<0.05 vs TAC cardiomyocytes. Statistical significance was evaluated using Student *t* test in A through E and 2-way ANOVA followed by post-hoc Fisher LSD test for multiple comparisons in F.

To determine whether Orai1 channels are involved in the pathological stress response, adult wild-type (WT) mice were subjected to transverse aortic constriction (TAC). After 5 weeks, mice showed clear left ventricular dysfunction and structural abnormalities compared with age-matched sham-operated mice, as assessed by morphometric and echocardiographic parameters (Tables 1 and 2). This was associated with an increase in Orai1 expression at both mRNA (Figure 1A) and protein (Figure 1B) in ventricular tissues. Orai1 antibody specificity was validated by western blot analysis using the Orai1 blocking peptide in the presence of the antibody (Figure Ia in the online-only Data Supplement), siRNA knockdown of Orai1 in human pulmonary arterial smooth muscle cells (Figure Ib in the online-only Data Supplement), knockout of Orai1 in fibroblasts isolated from Orai1^fl/fl^ mice exposed to ex vivo TAT-Cre (Figure Ic through If in the online-only Data Supplement), and by immunohistochemistry using the blocking peptide (Figure Ig and Ih in the online-only Data Supplement). Immunohistochemistry of ventricular sections from sham and TAC-operated mice showed Orai1 localization at the plasma membrane as indicated by colocalization with the surface membrane marker, wheat germ agglutinin (Figure 1C). Furthermore, we performed coimmunostaining of Orai1 with the cardiomyocyte marker, α-actinin, demonstrating Orai1 expression in cardiomyocytes (Figure Ii in the online-only Data Supplement). Analysis of the Orai1 fluorescence signal in isolated cardiomyocytes from sham-operated and TAC mice revealed a colocalization between Orai1 and wheat germ agglutinin (Figure 1D), which was increased after TAC (Figure 1E), whereas no striated pattern was found in comparison to α-actinin staining (Figure II in the online-only Data Supplement).

**Table 1.**
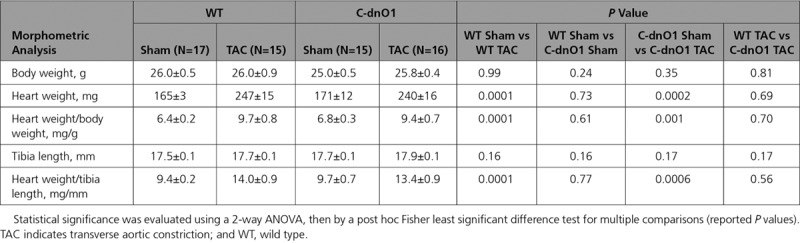
Morphometric Analysis Under Stressed Condition

**Table 2.**
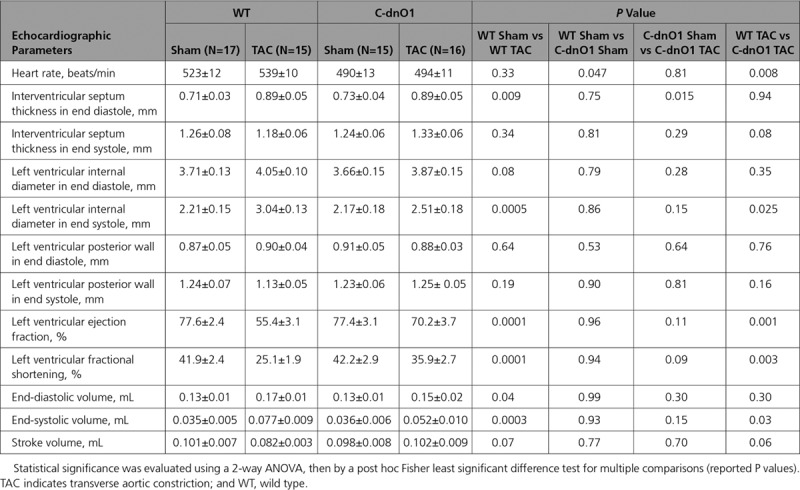
Cardiac Function Analyzed by Echocardiography Under Stressed Condition

TRPC6 (transient receptor potential canonical 6), Orai3 and STIM2 mRNA and protein levels were also upregulated (Figure III in the online-only Data Supplement) while TRPC1, -C3, -C4, -C5, STIM1 and Orai2 proteins were unchanged after TAC (Figure IV in the online-only Data Supplement). Freshly isolated ventricular cardiomyocytes from TAC mice showed a robust store-operated cation entry following Ca^2+^ store depletion compared with ventricular cardiomyocytes from sham-operated mice, as demonstrated by a faster reduction of fluorescence decay upon addition of Mn^2+^ in the presence of major Ca^2+^ entry pathways inhibitors (Figure 1F).

We developed a novel Orai1 small-molecule inhibitor, JPIII, based on the Synta66 scaffold (Figure Va in the online-only Data Supplement),^13^ which is known to be both potent and selective but with poor aqueous solubility limiting in vivo use. JPIII exhibited nanomolar potency against endogenous SOCE in HEK293 cells (Figure Va in the online-only Data Supplement) and was highly effective against SOCE in HEK293 cells transiently overexpressing Orai1/STIM1 (Figure Vb in the online-only Data Supplement) or a self-activating Orai1-SS chimera (Figure Vc in the online-only Data Supplement). Electrophysiological recordings in Orai1-transfected HEK293 cells also confirmed that JPIII is a potent Orai1 inhibitor with an IC_50_ of 244 ± 39 nM (Figure Vd in the online-only Data Supplement). Of note, JPIII did not modify activity of Orai3, TRPC5, TRPC6, TRPC4/C1, TRPC5/C1, or TRPM2 overexpressed in HEK293 cells (Figure VI in the online-only Data Supplement) suggesting Orai1 selectivity. Furthermore, in vitro assessment of pharmacokinetics (Figure VII in the online-only Data Supplement) supported suitability for in vivo delivery, although the relatively short half-life in murine liver microsomes suggested a need for continuous infusion during the in vivo studies.

JPIII (5 μM) significantly reduced depletion-induced cation entry in isolated ventricular cardiomyocytes from both sham- and TAC-operated mice (Figure 1F). Of note, the JPIII-sensitive cation entry in sham-operated mice was relatively small, consistent with a low level of Orai1 expression in adult rodent cardiomyocytes.^5,14,15^ It is important to note that JPIII treatment of the TAC cardiomyocytes reduced SOCE down to the level observed in the sham-operated mice. These results demonstrate that Orai1 is a major participant in the exacerbated store-operated cation entry response observed in cardiac hypertrophy.

### C-dnO1 Mice Display Preserved Ventricular Function and Ca^2+^ Handling Under Physiological Condition

**Figure 2.**
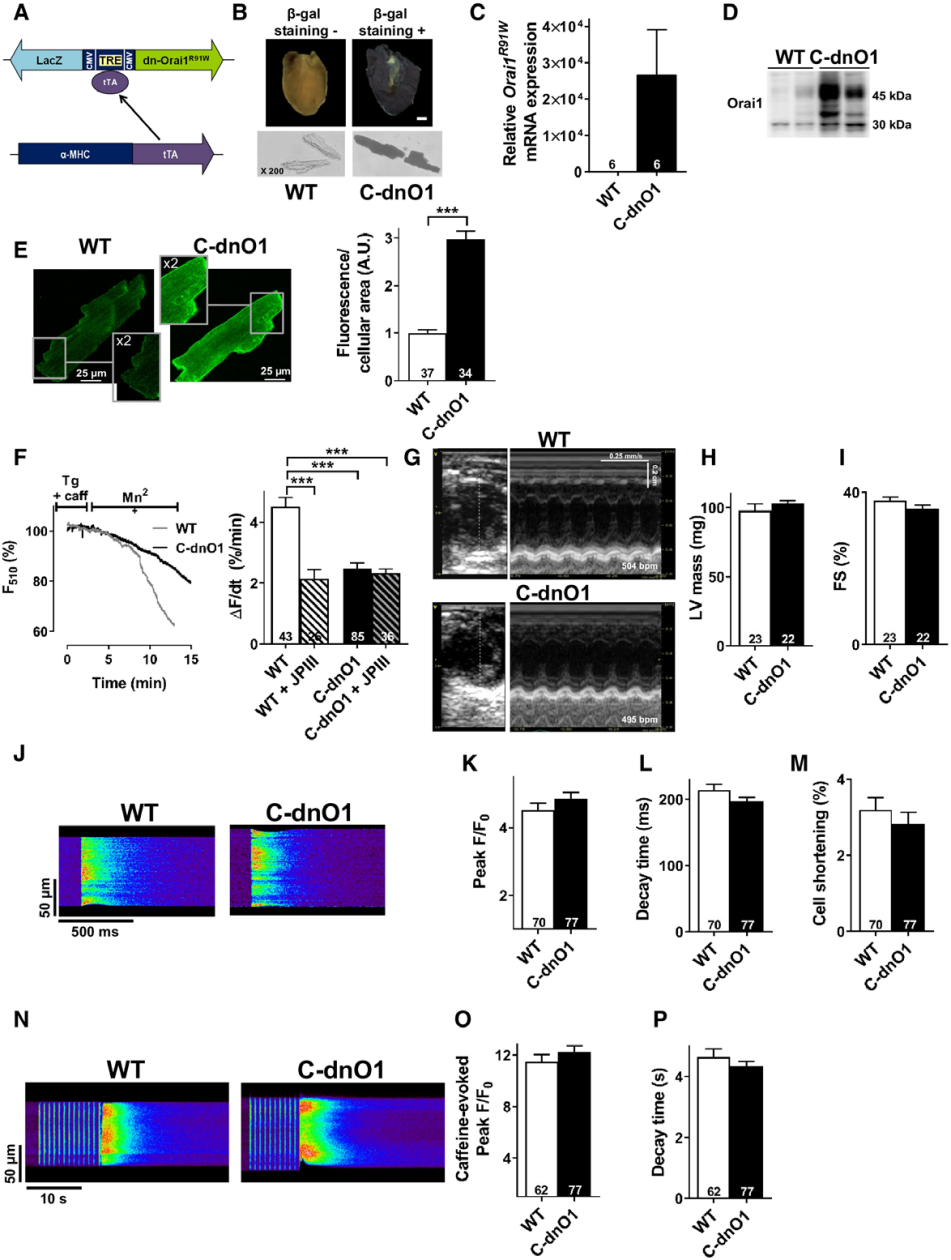
**Cardiomyocyte-specific human dominant-negative Orai1 channel expression in mice (C-dnO1) does not alter in vivo and in vitro cardiac function under physiological condition.** **A**, α-MHC-tTA/TetO-dn-Orai1^R91W^ mice, named C-dnO1, were obtained by crossing a transgenic mouse line that coexpresses human dn-Orai1^R91W^ and LacZ reporter under the control of a tetracycline response element (TRE) and another mouse line expressing tTA (tetracycline transactivator) gene under the regulation of a heart-specific α-MHC promoter. tTA binds the TRE promoter to drive the transcription of dn-Orai1^R91W^ and LacZ genes specifically in the cardiomyocytes. **B**, Blue-gal staining of a cross section of the heart and of adult ventricular cardiomyocytes from wild-type (WT; no color) and C-dnO1 mice (dark color). Scale bar, 1 mm. Magnification: X 200. **C**, Human Orai1 mRNA expression was determined by RT-qPCR in ventricle tissue from WT and C-dnO1 mice. mRNA levels were normalized to housekeeping genes and expressed as fold change of that determined in WT mice. N=6 animals. **D**, Representative western-blot of exogenous and endogenous Orai1 in ventricle tissue from WT and C-dnO1 mice. **E**, Representative confocal imaging (top panel) and quantification (bottom panel) of endogenous mouse Orai1 and human dn-Orai1 proteins in adult ventricular cardiomyocytes from WT and C-dnO1 mice. N=3 animals. n=34–37 investigated cells. Scale bar, 25 μm. Insets represent 2-fold magnification of main images. **F**, Representative fluorescence traces of the Fura-2 decay phase upon Mn^2+^ addition in cardiomyocytes from WT (grey trace) and C-dnO1 (black trace) mice (left panel) and quantification of store-operated cation entry (right panel) for cardiomyocytes from WT and C-dnO1 mice in presence or absence of 5 µM JPIII. N=3–5 animals. n=26–85 investigated cells. ***P<0.001 vs WT cardiomyocytes. **G**, Representative echocardiography left ventricular (LV) images in short axis and in M-mode from WT and C-dnO1 mice. Averaged LV parameters, at baseline, obtained from analysis of echocardiograms: LV mass (**H**) and cardiac fractional shortening (FS, %) (**I**) in WT and C-dnO1 mice. N=22–23 animals. **J**, Representative line-scan of [Ca^2+^]_i_ transients in cardiomyocytes field-stimulated at 1 Hz and loaded with Fluo-4 from WT and C-dnO1 mice. **K**, Average of [Ca^2+^]_i_ transients amplitude (peak F/F_0_). **L**, Average of [Ca^2+^]_i_ transients decay time constant (ms). **M**, Average of the % of cell shortening. **N**, Representative line-scan of caffeine-evoked [Ca^2+^]_i_ transients. **O**, Average of caffeine-evoked SR Ca^2+^ load amplitude (peak F/F_0_). **P**, Average of caffeine-evoked SR Ca^2+^ load decay time constant (s). N=3–4 animals. n=62–77 investigated cells. Statistical significance was evaluated using Student *t* test except in F where 2-way ANOVA followed by post-hoc Fisher LSD test for multiple comparisons was used.

To examine the pathophysiological role of Orai1 in the heart, we generated a transgenic mouse line coexpressing the human dominant-negative Orai1 channel (missense mutation R91W)^16^ and LacZ reporter using the Tet-Off inducible system (TetO-dn-Orai1^R91W^). To allow cardiomyocyte-specific expression, the TetO-dn-Orai1^R91W^ strain was crossed with the α-MHC-tTA strain (Figure 2A), which showed heart specific expression in both ventricle and atrium compartment but not in other tissues.^17,18^ The double transgenic animal is hereafter referred to as C-dnO1. C-dnO1 offspring showed the expected Mendelian ratio and developed normally. No obvious in vivo or in vitro cardiac function differences were observed in α-MHC-tTA or TetO-dn-Orai1^R91W^ mice compared with WT littermates, and then pooled as WT. Heart tissue and freshly isolated ventricular cardiomyocytes from C-dnO1 mice displayed blue-gal staining, which was absent in heart and cardiomyocytes from WT littermates (Figure 2B). Similarly, human Orai1 mRNA was detected by quantitative reverse transcription polymerase chain reaction only in ventricular tissue from C-dnO1 mice (Figure 2C). We further confirmed the overexpression of exogenous Orai1 with the classical multiple band pattern of human Orai1 protein in ventricle tissue by western blot (Figure 2D).^19,20^ In isolated cardiomyocytes, Orai1 immunostaining revealed a sarcolemmal profile in WT cardiomyocytes, which was more pronounced in C-dnO1 cells (Figure 2E). Any potential compensation by other store-operated Ca^2+^ channel components (TRPC1 to -C6, STIM1-2 and Orai3 protein) was observed (Figure VIII in the online-only Data Supplement). Cardiomyocytes from C-dnO1 mice or from WT mice treated with 5 µM JPIII displayed reduced store-operated cation entry compared with WT cells (Figure 2F). It is important to note that Orai1 inhibition by dn-Orai1^R91W^ expression and JPIII application was not cumulative, indicating a complete blocking effect of dn-Orai1^R91W^ mutant and equivalence between the two strategies. These data demonstrated that the Orai1^R91W^ mutant was successfully expressed in heart and that selective SOCE inhibition was achieved.

To characterize the functional consequences of Orai1 blockade on in vivo cardiac electromechanical function, echocardiography on anesthetized mice and electrocardiograms by telemetry on conscious mice were performed at 2 to 3 months of age. Compared with WT littermate mice, C-dnO1 mice did not show any signs of cardiac dysfunction or alteration in overall phenotype, as assessed by morphometric (heart weight/body weight and heart weight/tibia length ratios) and echocardiographic measurements (Tables 3 and 4 and Figures 2G and 2I). It is notable that C-dnO1 mice showed normal and conserved left ventricular (LV) contractility performance as measured by LV fractional shortening (Figure 2I). This was also observed in C-dnO1 mice at 6 months of age (Figure IX in the online-only Data Supplement). Of note, the tendency to observe lower heart rate in anesthetized C-dnO1 mice would point a slight improvement in systolic function in these mice compared with WT mice. In addition, cardiac electrical activity evaluated by telemetric electrocardiograms from awake mice over 24 hours did not show statistical differences in heart rate, in conduction intervals, nor in ventricular activity (PR, QRS, and QT intervals) between groups (Figure X in the online-only Data Supplement).

**Table 3.**
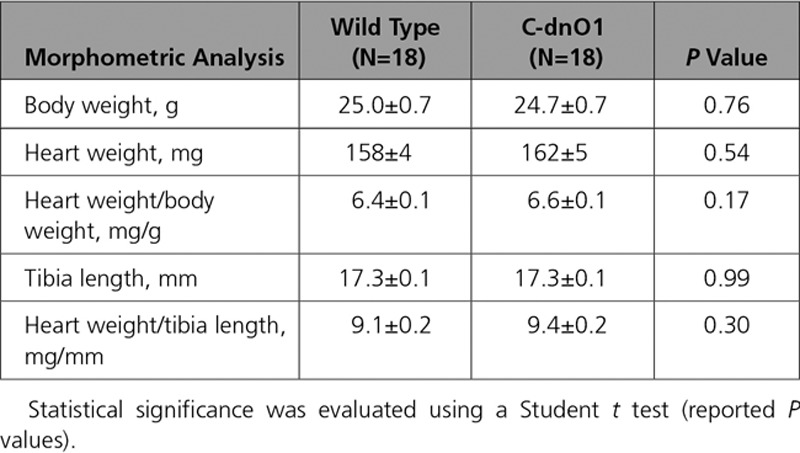
Morphometric Analysis Under Basal Condition

**Table 4.**
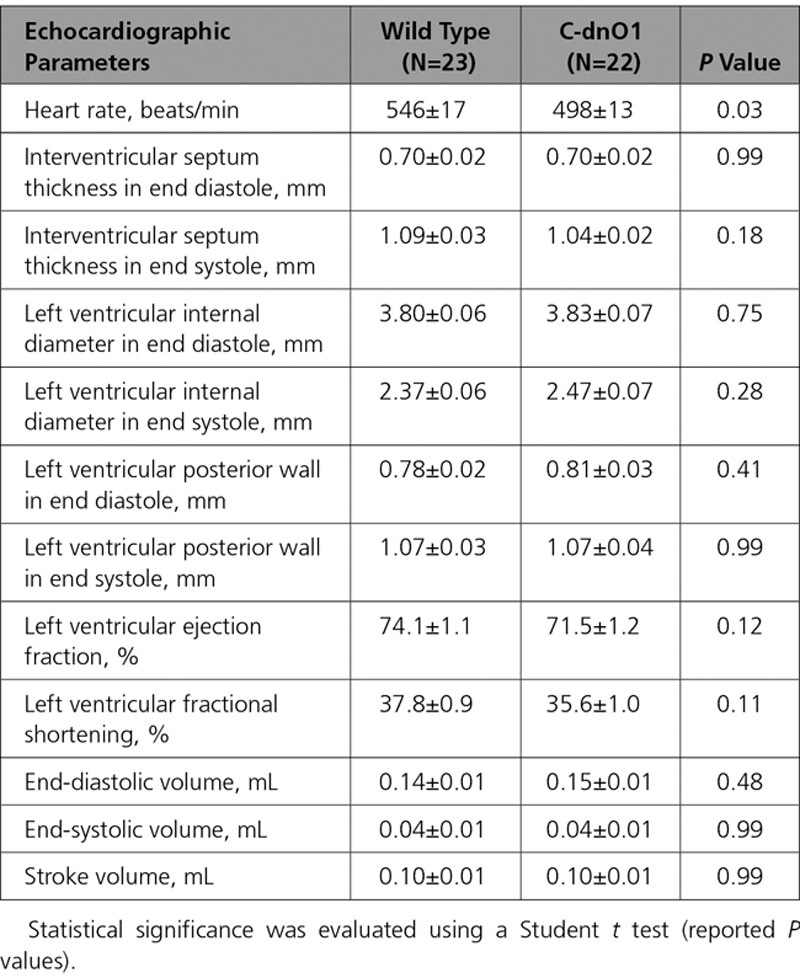
Cardiac Function Analyzed by Echocardiography Under Basal Condition

Consistent with these whole animal observations, no major alterations were observed at cellular level. Freshly isolated LV cardiomyocytes from WT littermates and C-dnO1 mice did not present signs of cellular hypertrophy as assessed by cellular area and membrane capacitance measurements (Table 5). Action potential waveforms, recorded using the whole-cell patch-clamp technique, were similar in both groups (Table 5). Likewise, we found no differences in cellular Ca^2+^ homeostasis indicated by analysis of paced-[Ca^2+^]_i_ transients or caffeine-induced [Ca^2+^]_i_ transients using confocal line scan imaging of Fluo-4 loaded cardiomyocytes from WT littermates and C-dnO1 mice (Figures 2J through 2P). Similarly, no effects on the amplitude of [Ca^2+^]_i_ transients, cell shortening, and the sarcoplasmic reticulum (SR) Ca^2+^ load were observed in isolated ventricular cardiomyocytes from WT mice before and after perfusion with 5 µM of JPIII and of isolated ventricular cardiomyocytes from mice treated for 3 weeks with JPIII (500 ng/kg/min; Figure XI in the online-only Data Supplement). Moreover, no differences in RyR activity, measured as frequency and biophysical characteristics of spontaneous Ca^2+^ sparks (Figures XIIa through XIIe in the online-only Data Supplement), or in diastolic Ca^2+^ levels, determined by Fura-2 ratio (ratio: 340/380; Figure XIIf in the online-only Data Supplement), were observed between cardiomyocytes of C-dnO1 mice and WT littermates.

**Table 5.**
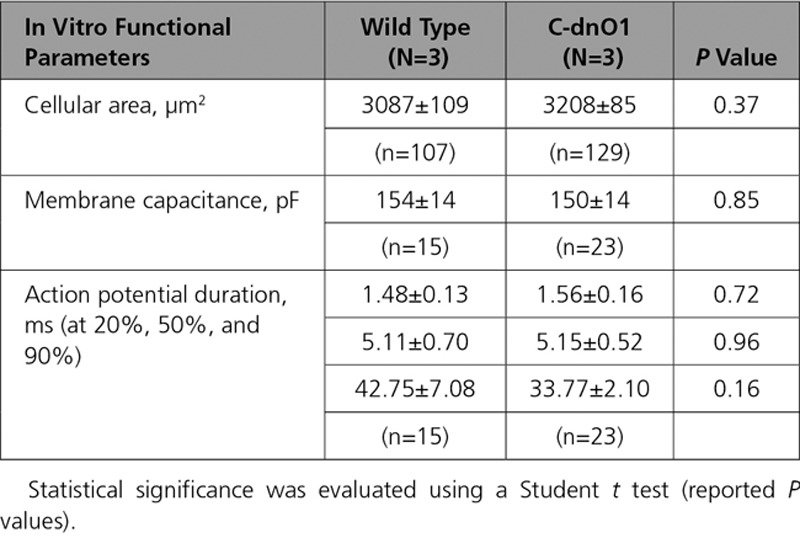
In Vitro Functional Parameters Under Basal Condition

Taken together, these data suggest that the inhibition of Orai1, under physiological conditions, does not directly modulate cardiac excitation-contraction coupling and does not cause any physiological consequences.

### Loss of Cardiomyocyte Orai1 Function Protects the Heart From Pressure Overload-Induced Systolic Dysfunction

**Figure 3.**
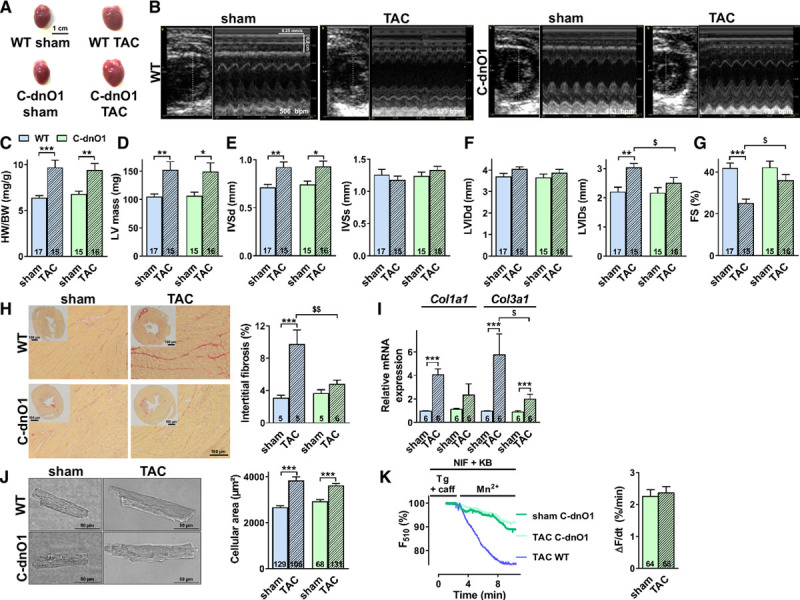
**Depressed systolic function observed in wild-type mice 5 weeks after TAC is prevented in C-dnO1 mice.** **A**, Representative whole hearts from wild-type (WT) and C-dnO1 sham or transverse aortic constriction (TAC)-operated mice. Scale bar, 1 cm. **B**, Representative echocardiography images of LV in short-axis and in M-mode from WT and C-dnO1 sham or TAC mice. **C**, Morphometric analysis from WT and C-dnO1 sham or TAC mice. N=15–16 animals. **D** through **G**, LV parameters obtained from analysis of echocardiograms: LV mass (**D**), interventricular septal thickness at end of diastole (IVSd) and systole (IVSs) (**E**), LV internal diameter at end of diastole (LVIDd) and systole (LVIDs) (**F**) and cardiac FS % (**G**) in WT and C-dnO1 mice after TAC. N=15–17 animals. *P<0.05, **P<0.01, ***P<0.001 vs respective sham mice. ^$^*P*<0.05 vs WT TAC mice. **H**, Left panel: representative images of interstitial collagen deposition in heart tissue sections stained with Picrosirius red from WT and C-dnO1 sham or TAC mice. Scale bar, 100 µm. Right panel: analysis of interstitial collagen staining as a percentage of tissue area. N=5–6 animals. ***P<0.001 vs sham mice. ^$$^*P*<0.01 vs WT TAC mice. **I**, Type I collagen (*Col1a1*) and type III collagen (*Col3a1*) mRNA expression was determined by RT-qPCR in ventricle tissue from WT and C-dnO1 sham or TAC mice. mRNA levels were normalized to housekeeping genes and expressed as fold change of that determined in WT sham mice. N=6 animals. ***P<0.001 vs respective sham mice. ^$^*P*<0.05 vs WT TAC mice. **J**, Left panel: representative conventional microscopy images of WT and C-dnO1 cardiomyocytes from sham and TAC mice. Scale bar, 50 μm. Right panel: cell size (µm^2^) measured by planimetry. N=3–4 animals. n=68–131 investigated cells. ***P<0.001 vs respective sham mice. **K**, Representative fluorescence traces (left panel) and quantification (right panel) of the Fura-2 decay phase upon Mn^2+^ addition in cardiomyocytes from C-dnO1 sham and TAC mice. N=3 animals. n=64–68 investigated cells. Statistical significance was evaluated using 2-way ANOVA followed by post-hoc Fisher LSD test for multiple comparisons, except in K where Student *t* test was used.

To determine the functional relevance of Orai1 upregulation in the pathogenesis of cardiac hypertrophy, adult C-dnO1 mice were subjected to pressure overload by TAC. After 5 weeks of TAC, WT and C-dnO1 mice showed a similar degree of cardiac hypertrophy as revealed by morphometric and echocardiographic analysis (Tables 1 and 2, Figures 3A through 3F). C-dnO1 mice showed a similar increase in LV mass (estimated by Penn formula, Figure 3D) and in interventricular septum thickness in diastole (Figure 3E) compared with WT littermates. Moreover, C-dnO1 mice showed similar induction of hypertrophic marker gene expression such as *Nppa* and *Acta1* after 5 weeks of TAC (Figure XIII in the online-only Data Supplement). It is interesting to note that Orai3 and STIM2 upregulation was reduced in C-dnO1 after TAC (Figures IIId and IIIf in the online-only Data Supplement). However, echocardiographic analysis revealed protection from the development of systolic dysfunction in C-dnO1 mice compared with WT littermates. A significant 40% decrease in LV fractional shortening (Figure 3G) associated with markedly increased LV end systolic volume and decreased stroke volume (Table 2) was observed after TAC in WT mice. This was only reduced by 16% in C-dnO1 mice (Figure 3G) with preserved systolic and stroke volumes (Table 2).

It is important to note that we observed less interstitial collagen deposition using Picosirius red staining in heart sections from C-dnO1 versus WT mice compared with their respective sham-operated counterparts (Figure 3H). This is correlated in C-dnO1 mice with less induction of fibrillar type III collagen (*Col3a1*) transcripts after 5 weeks of TAC (Figure 3I).

At a cellular level, planimetric cell area measurements (Figure 3J) showed similar hypertrophy in isolated ventricular cardiomyocytes from WT littermates and C-dnO1 mice after TAC. Moreover, the prolongation of action potential duration (APD), electrophysiological stigmata of cardiac hypertrophy, is similarly observed in WT and C-dnO1 mice after TAC (WT sham cardiomyocytes: APD [in ms] at 20%: 1.44±0.08, at 50%: 5.02±0.47, at 90%: 34.5±6.0 [n=11]; WT TAC cardiomyocytes: at 20%: 2.89±1.0, at 50%: 11.89±4.26, at 90%: 76.77±9.84 [n=19]; C-dnO1 TAC cardiomyocytes: APD at 20%: 3.55±1.48, at 50%: 10.71±3.89, at 90%: 91.07±25.79 [n=11]), *P*<0.001 versus WT sham. However, the exacerbated store-operated cation entry found in WT after TAC (Figure 1) was abolished in C-dnO1 mice (Figure 3K).

Collectively, these results suggest that inhibiting Orai1 function in the heart could be beneficial after insult by protecting it against systolic dysfunction.

### Loss of Cardiomyocyte Orai1 Function Prevents the Ca^2+^ Cycling Mishandling and Pyk2/MEK/ERK Pathway Activation After TAC

**Figure 4.**
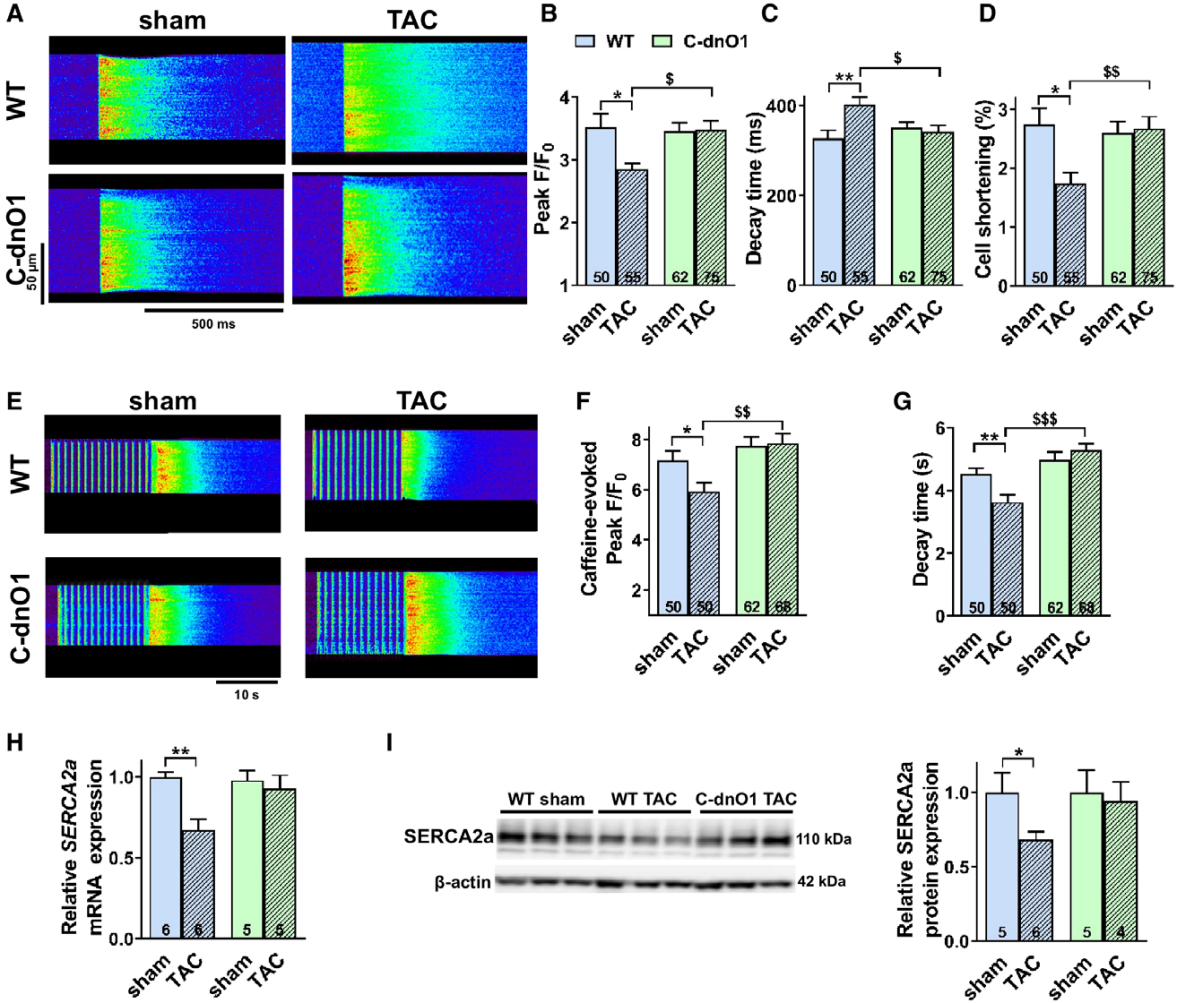
**The Ca^2+^ cycling mishandling observed in wild-type mice 5 weeks after TAC is restored in C-dnO1 mice.** **A**, Representative line-scan of [Ca^2+^]_i_ transients in cardiomyocytes field-stimulated at 1 Hz and loaded with Fluo-4 from wild-type (WT) and C-dnO1 sham or transverse aortic constriction (TAC) mice. **B**, Average of [Ca^2+^]_i_ transients amplitude (peak F/F_0_). **C**, Average of [Ca^2+^]_i_ transients decay time constant (ms). **D**, Average of the % of cell shortening. **E**, Representative line-scan of caffeine-evoked [Ca^2+^]_i_ transients. **F**, Average of caffeine-evoked SR Ca^2+^ load amplitude (peak F/F_0_). **G**, Average of caffeine-evoked SR Ca^2+^ load decay time constant (s). N=3–4 animals. n=50–75 investigated cells. *P<0.05, **P<0.01 vs WT sham mice. ^$^*P*<0.05, ^$$^*P*<0.01, ^$$$^*P*<0.001 vs WT TAC mice. **H**, *SERCA2a* mRNA expression was determined by RT-qPCR in ventricle tissue from WT and C-dnO1 sham or TAC mice after 5 weeks. mRNA levels were normalized to housekeeping genes and expressed as fold change of that determined in sham mice. N=5–6 animals. **P<0.01 vs WT sham mice. **I**, Representative western-blot and quantification of SERCA2a protein in ventricle tissue from sham and TAC mice. Protein levels were normalized by β-actin and expressed as fold change of that determined in sham mice. N=4–6 animals. *P<0.05 vs WT sham mice. Statistical significance was evaluated using 2-way ANOVA followed by post-hoc Fisher LSD test for multiple comparisons.

To obtain cellular mechanistic insights, we next examined the modulation of Ca^2+^ signaling after TAC in WT and C-dnO1 mice. Figure 4A illustrates confocal line-scan images of steady-state paced-[Ca^2+^]_i_ transients of isolated ventricular cardiomyocytes from the different groups. After TAC, the [Ca^2+^]_i_ transients amplitude (Figure 4B) was significantly decreased together with a prolonged decline (Figure 4C) and a reduction in cell shortening (Figure 4D) in WT cardiomyocytes. All these TAC-induced alterations were prevented in cardiomyocytes from C-dnO1 mice (Figures 4A through 4D). The reduction in [Ca^2+^]_i_ transients and myocyte shortening in WT mice after TAC were attributable to a decrease in SR Ca^2+^ load, as assessed by both peak decrease and decay deceleration of the caffeine-induced [Ca^2+^]_i_ transients (Figures 4E through 4G). It is remarkable that cardiomyocytes from C-dnO1 mice fully rescued this phenotype after TAC (Figures 4E through 4G). Consistent with the Orai1 upregulation relevance after hypertrophic insult, the F340/380 ratio recorded with Fura-2 is decreased after TAC when Orai1 is inhibited (Figure XIV in the online-only Data Supplement).

Consistent with the decreased SR Ca^2+^ load, the reduction in SERCA2a at mRNA and protein levels in WT ventricles was prevented in C-dnO1 mice after TAC (Figure 4H and 4I and Figure XVa in the online-only Data Supplement).

**Figure 5.**
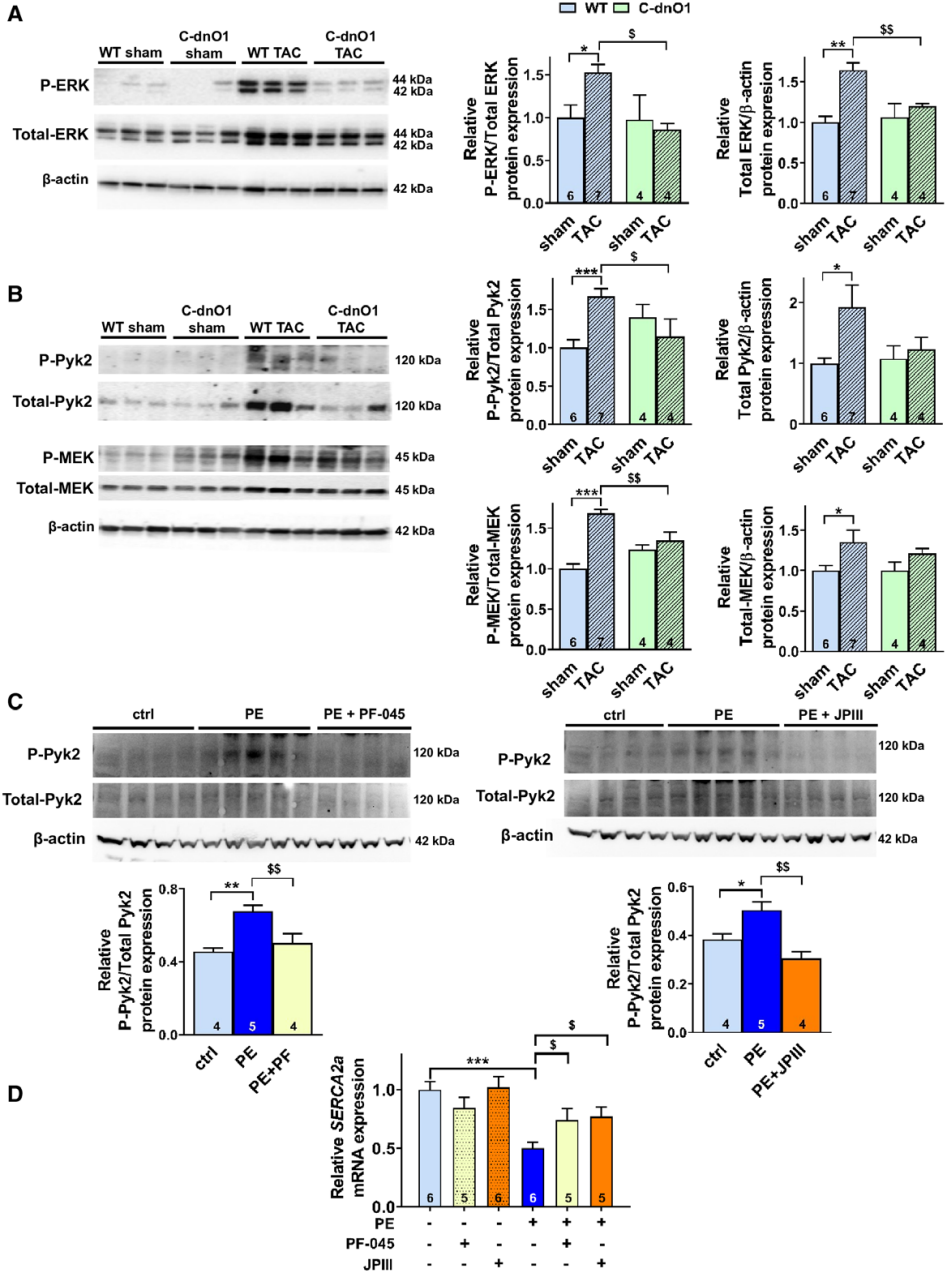
**Increased MEK/ERK and Pyk2 activity observed in wild-type mice 5 weeks after TAC is blunted in C-dnO1 mice.** **A** and **B**, Representative western-blots and quantifications of P-ERK1/2 and Total-ERK1/2 (**A**),of P-Pyk2 and Total-Pyk2 (**B**) and of P-MEK and Total-MEK (**B**) in ventricle tissue from sham and transverse aortic constriction (TAC) mice. Protein levels were normalized by β-actin and expressed as fold change of that determined in sham mice. N=4–7 animals. *P<0.05, **P<0.01, ***P<0.001 vs wild type (WT) sham mice. ^$^*P*<0.05, ^$$^*P*<0.01 vs WT TAC mice. **C**, Representative western-blots and quantifications of P-Pyk2 and Total-Pyk2 in ctrl or phenylephrine-treated neonatal rat ventricular cardiomyocytes (NRVMs) in presence of Pyk2 inhibitor (PF-04520440) or JPIII. Phosphorylation levels were normalized by Total-Pyk2 expression and expressed as fold change of that determined in ctrl NRVMs. **D**, Relative *SERCA2a* mRNA expression was determined by RT-qPCR in ctrl or phenylephrine-treated NRVMs in presence of PF-04520440 or JPIII. mRNA levels were normalized to housekeeping genes and expressed as fold change of that determined in ctrl NRVMs. *P<0.05, **P<0.01, ***P<0.001 vs ctrl NRVMs. ^$^*P*<0.05, ^$$^*P*<0.01 vs phenylephrine-treated NRVMs. Statistical significance was evaluated using 2-way ANOVA followed by post-hoc Fisher LSD test for multiple comparisons except in C where 1-way ANOVA was used.

No changes in CaMKII and calcineurin activities, Ca^2+^-dependent pathways involved in the pathogenesis of cardiac hypertrophy, as well as their targets (RyR and PLB phosphorylation states) were found after TAC in both conditions (Figure XVb through XVe in the online-only Data Supplement). However, as previously demonstrated in neonatal cardiomyocytes,^4^ we found a significant increase in ERK1/2 activity and expression after TAC, which was totally prevented in ventricles from C-dnO1 mice (Figure 5A). Western blot analysis of the upstream signaling pathway showed that TAC-induced increases in MEK and Pyk2 (Figure 5B) expression and phosphorylation were prevented in C-dnO1 mice (Figure 5B), suggesting that a Pyk2/MEK/ERK cascade is associated with the observed Orai1 inhibition-dependent protective effects.

Similarly and consistent with previous studies,^21–23^ in neonatal rat ventricular cardiomyocytes exposed for 48 hours to 100 µM phenylephrine, which induced increases in prohypertrophic genes such as *Nppa* and *Nppb* (Figure XVIa in the online-only Data Supplement) and in Orai1 expression (Figure XVIb and XVIc in the online-only Data Supplement), cotreatment during the past 24 hours with a Pyk2 inhibitor PF-04520440 (5 µM) or JPIII (5 µM) prevented the increased Pyk2 phosphorylation (Figure 5C) and reduced the downregulation of *SERCA2a* mRNA expression (Figure 5D). In this in vitro model, the phenylephrine-induced increased ERK phosphorylation was not changed after Pyk2 and Orai1 inhibition (Figure XVId in the online-only Data Supplement).

Overall, our results suggest that preserved cardiac inotropy function by cardiomyocyte Orai1 inhibition is related to prevention of altered Ca^2+^ cycling and SERCA2a expression associated with Pyk2 and/or MEK/ERK pathway.

### Orai1 Inhibition by JPIII Infusion Protects the Heart From Pressure Overload-Induced Systolic Dysfunction

Next, we aimed to determine whether the effect of the C-dnO1 construct could be recapitulated by a translationally relevant small molecule inhibitor approach. The JPIII pharmacokinetic data (Figure VII in the online-only Data Supplement) supported the suitability of JPIII for this purpose. The JPIII stock solution was detectable by liquid chromatography-mass spectrometry as a single ion peak at 321.2 m/z with a characteristic UV chromatogram for the extracted peak (Figure XVIIa in the online-only Data Supplement). JPIII was stable at physiological temperature for 28 days and not degraded by the material used in the osmotic mini-pumps compared with the stock solution alone (Figures XVIIb and XVIIc in the online-only Data Supplement). Dose finding studies (Figure XVIId in the online-only Data Supplement) demonstrated detection of JPIII in serum 15 minutes after administration of 0.72 mg/kg/day (equivalent to an infused dose of 500 ng/kg/min via osmotic mini-pump). Pilot studies dosing C57BL6/J mice with JPIII at 500 ng/kg/min via subcutaneous osmotic mini-pump infusion for 28 days demonstrated no adverse effects on the animals. Animal weights (Figure XVIIe in the online-only Data Supplement), systolic and diastolic blood pressure measured in awake restrained mice by tail cuff plethysmography (Figure XVIIf in the online-only Data Supplement), and the heart weight–body weight ratio (Figure XVIIg in the online-only Data Supplement) were similar between the JPIII and vehicle infused groups. Blood sampling from the saphenous vein on day 14 of the infusion confirmed presence of JPIII in the serum (Figure XVIIh in the online-only Data Supplement) and explant of the pumps at the end of the experiment (Figure XVIIh inset in the online-only Data Supplement) confirmed successful delivery. A toxicology screening study performed in CD-1 outbred mice infused with JPIII (500 ng/kg/min) or vehicle via osmotic mini-pump for 28 days showed no major toxic effect on the animals; in particular no changes in total or differential white cell counts were observed (Figure XVIII in the online-only Data Supplement). JPIII is therefore a suitable novel tool compound for testing the hypothesis in murine models that can be effectively delivered by osmotic mini-pump infusion.

**Figure 6.**
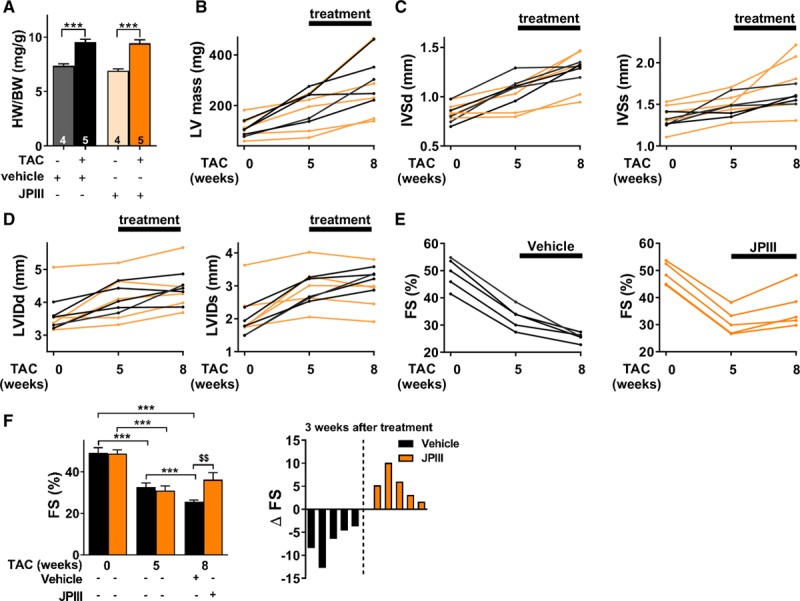
**Orai1 inhibition by JPIII protects the heart from pressure overload-induced ventricular systolic dysfunction.** **A**, Morphometric analysis from sham and transverse aortic constrction (TAC)-operated mice. N=4–5 animals. ***P<0.001 vs respective sham mice. Statistical significance was evaluated using 2-way ANOVA followed by post-hoc Fisher LSD test for multiple comparisons. **B** and **C**, Time-dependent effect of vehicle- and JPIII-treatment on LV mass (**B**) and interventricular septal thickness at end diastole and systole (**C**) in sham and TAC mice. N=5 animals. **D**, Time-dependent effect of vehicle- and JPIII-treatment on LV internal diameter at end diastole and systole in sham and TAC mice. N=5 animals. **E** and **F**, Time-dependent effect of vehicle- and JPIII-treatment on LV FS (%) in sham (left panel) and TAC-operated (right panel) mice. N=5 animals. Statistical significance was evaluated using 2-way repeated measures ANOVA. ***P<0.001 vs before TAC or 5 weeks after TAC. ^$$^*P*<0.01 vs vehicle-treated mice after 8 weeks of TAC. **F**, Right panel: % of LV FS for each mice presented in E obtained by subtracting FS after and before treatment. FS indicates fractional shortening; interventricular septal thickness at end of diastole; IVSd, interventricular septal thickness at end of diastole; IVSs; interventricular septal thickness at end of diastole systole; JPIII, 4-(2,5-dimethoxyphenyl)-N-[(pyridin-4-yl)methyl] aniline; LV, left ventricular; and LVIDd, LV internal diameter at end of diastole.

**Figure 7.**
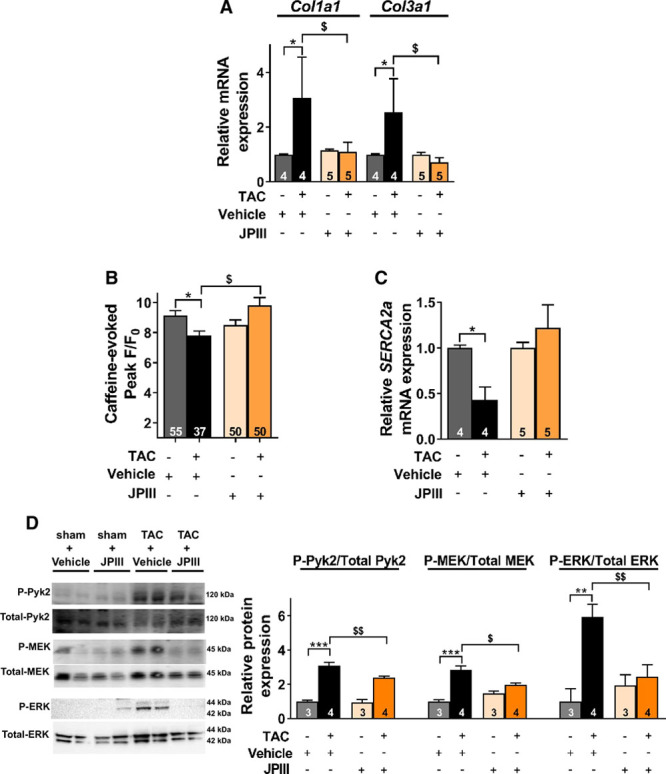
**Orai1 inhibition by JPIII protects the heart from pressure overload-induced ventricular Ca^2+^ mishandling.** **A**, Type I collagen (*Col1a1*) and type III collagen (*Col3a1*) mRNA expression was determined by RT-qPCR in ventricle tissue from WT sham or TAC mice treated or not with JPIII. mRNA levels were normalized to housekeeping genes and expressed as fold change of that determined in sham mice. N=4–5 animals. *P<0.05 vs respective sham mice, ^$^*P*<0.05 vs vehicle-treated TAC mice. **B**, Average of caffeine-evoked SR Ca^2+^ load amplitude (peak F/F_0_). N=3–4 animals. n=37–55 investigated cells. *P<0.05 vs sham mice, ^$^*P*<0.05 vs vehicle-treated TAC mice. **C**, *SERCA2a* mRNA expression was determined by RT-qPCR in ventricle tissue from sham or TAC mice treated or not with JPIII. mRNA levels were normalized to housekeeping genes and expressed as fold change of that determined in sham mice. N=4–5 animals. *P<0.05 vs sham mice. **D**, Representative western-blots and quantification of phosphorylated over total forms of Pyk2, MEK and ERK1/2 in ventricular cardiomyocytes from sham and TAC mice treated or not with JPIII, expressed as fold change of that determined in vehicle-treated sham mice. N=3–4 animals. **P<0.01, ***P<0.001 vs sham mice. *P<0.05, ^$$^*P*<0.01 vs vehicle-treated TAC mice. Statistical significance was evaluated using 2-way ANOVA followed by post-hoc Fisher LSD test for multiple comparisons. TAC indicates transverse aortic constriction.

To confirm the potential therapeutic benefit of Orai1 inhibition after hypertrophic insult, WT mice were subjected to 5 weeks TAC and then, randomly assigned to constant delivery of vehicle (DMSO) or JPIII (at 500 ng/kg/min) by osmotic mini-pump for 3 weeks. This approach showed that cardiac hypertrophy was not affected in JPIII-treated mice compared with vehicle-treated WT animals (Figure 6A through 6D, Tables 6 and 7). However, the progressive decline in systolic function measured by LV fractional shortening (%) was blunted after JPIII treatment (Figure 6E and 6F, Table 7). Of note, vehicle or JPIII treatment did not alter heart morphology or ventricular function of sham-operated animals (Table 6 and 7, Figure XIX in the online-only Data Supplement). However, we observed complete prevention of the increase in fibrillar type I and III collagens (*Col1a1* and *Col3a1*) transcripts in JPIII-treated hearts compared with vehicle-treated hearts after TAC (Figure 7A).

**Table 6.**
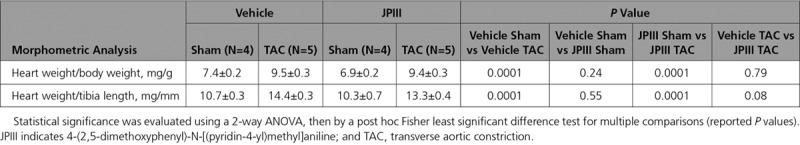
Morphometric Analysis 8 Weeks After TAC in Vehicle and JPIII-Treated Mice

At the cellular level, this effect was associated with preserved SR Ca^2+^ load (Figure 7B) correlating with rescue of SERCA2a expression (Figure 7C) and reduced Pyk2, MEK and ERK1/2 activities (Figure 7D) from JPIII-treated mice after TAC. The data suggest that JPIII, like C-dnO1, protects against specific aspects of systolic dysfunction.

## Discussion

Our results suggest that genetic or pharmacologic inhibition of cardiomyocyte Orai1 is not instrumental in regulating excitation-contraction coupling and cardiac function in the adult mouse heart but that it is cardioprotective against pressure overload-induced systolic dysfunction without affecting cardiac hypertrophy itself. These beneficial effects were associated with a reduction in the extent of interstitial fibrosis and prevention of Ca^2+^ homeostasis alterations. In addition, we showed that selective Orai1 channel blockade is a potential effective therapeutic for systolic dysfunction after chronic pressure overload.

The existence and contribution of SOCE is well established in the developing heart for regulating diastolic Ca^2+^ homeostasis and cardiac growth.^7^ Orai1 expression is low in adult mouse heart and the functional significance of SOCE in the normal adult heart is not well defined. Under physiological conditions, we found that cardiomyocyte-specific functional inhibition of Orai1 channels in C-dnO1 mice had little impact, with normal LV contractility performance until 6 months of age, similar to what has previously been demonstrated with global heterozygous Orai1^+/-^ mice^10^ alongside normal electrical function. At a cellular level, no alterations of action potential or Ca^2+^ homeostasis were observed although the SOCE was significantly decreased. Consistent with data obtained in C-dnO1 mice, acute or chronic pharmacological Orai1 inhibition by JPIII also has no impact on excitation-contraction coupling. Thus, Orai1 activity makes no contribution to the electromechanical function of normal ventricular cardiomyocytes. This is consistent with previous results using a nonselective Orai1 blocker or Orai1 knock-down in feline and rat hearts.^24–26^ Our results on excitation-contraction coupling are not surprising given the slow Orai1 activation kinetic (10s to 100s) because of the need for STIM translocation in non-excitable cells.^27,28^ This is not compatible to fast excitation-contraction coupling (ms ranged) even if ultra-rapid (in a range of ms) activation of SOCE has been described in skeletal muscle.^29^ On the other hand, double TRPC1/C4 KO mice exhibited lower diastolic and systolic Ca^2+^ concentrations, under basal conditions.^30^ By contrast, enhanced cardiac pump function was found in cardiac specific dn-TRPC4 mice with increased myocyte fractional shortening and peak [Ca^2+^]_i_ transients^31^ suggesting that TRPC subfamily, but not Orai1, fine tunes Ca^2+^ cycling in beating adult mice cardiomyocytes.

Our combination of genetic and pharmacologic Orai1 inhibition approaches revealed a contribution of cardiomyocyte Orai1 channels in pressure overload-induced systolic dysfunction. Consistent with previous studies,^5,12^ Orai1 expression was increased after 5 weeks of TAC, exacerbating SOCE. Contrasting with compensated hypertrophic animal models that lack alteration of systolic function as assessed by the absence of LV fraction shortening modification,^12,24,32^ our model of pressure overload-induced cardiac hypertrophy associated with ventricular dysfunction produces a more severe phenotype and lacks upregulation of STIM1 expression. Nevertheless, we found a significant upregulation of STIM2 after TAC. This upregulation is also reduced in C-dnO1 mice after TAC. STIM2 is known to participate to the SOCE machinery^33,34^ and have a crucial function in inducing the activated conformation of STIM1 causing STIM1/Orai1 coupling and enhancement of Orai1 function.^35^ This allows us to suggest that enhanced Orai1-dependent Ca^2+^ entry after hypertrophic insult is potentially dependent on STIM2, directly or indirectly, which is a new concept worthy of future further investigation.

The direct contribution of Orai1-dependent Ca^2+^ entry to the hypertrophic process is still under debate. Indeed, in vitro data showed that neurohormonal stimulation increased Orai1 expression^11^ and that Orai1 silencing completely abrogated the hypertrophic signal by inhibiting CaMKII and the ERK1/2 signaling pathway^4,11^ highlighting a protective effect of inhibiting Orai1 after hypertrophic insults. However, global heterozygous Orai1^+/-^ mice submitted to TAC had greater dilation of left ventricle and decline in ventricular function than WT mice suggesting a deleterious effect of constitutive Orai1 haploinsufficiency.^10^ Here, while the pathological changes in ventricular geometry were similar between WT and cardiomyocyte C-dnO1 mice after TAC, C-dnO1 mice exhibited preserved systolic function. The fact that inhibiting Orai1 function in the heart could be beneficial after insult clarifies the situation and provides an important breakthrough, suggesting that the global Orai1^+/-^ is related to either constitutive deletion during development or systemic alteration and not to cardiac specific alteration in the adult.

In our conditions, inhibiting Orai1 did not prevent the development of cardiac hypertrophy. This may be because of an increased TRPC6 expression following TAC, as TRPC6 has been identified as a key player influencing the development of hypertrophy and heart disease by inducing calcineurin/NFAT signaling.^36–38^ Supportive of this concept, increased calcineurin activity after TAC was similar in WT and C-dnO1 mice suggesting that Orai1 is not instrumental in a calcineurin-dependent hypertrophic process in this pathology. The concept that prevention of hypertrophy is beneficial in cardiac disease is now controversial. Indeed, some reports showed that hypertrophy blockade may be detrimental.^39^ In the same way, cardiac STIM1 silencing in mice impairs adaptive hypertrophy and promotes the transition to HF.^40^

Moreover, we highlighted that the preserved ventricular function observed in C-dnO1 mice is related to maintaining normal Ca^2+^ cycling. Indeed, all Ca^2+^ signaling alterations observed in cardiomyocytes from WT TAC animals, which includes decreased [Ca^2+^]_i_ transient amplitude, slowing of the systolic [Ca^2+^]_i_ transients associated with lower Ca^2+^ SR load and decreased level of SERCA2a expression and depressed cellular contractility were prevented in ventricular cardiomyocytes from C-dnO1 mice.

Analysis of upstream signaling pathways revealed that a sustained increase in intracellular Ca^2+^ through Orai1 is associated with Pyk2 (a member of the FAK family of protein tyrosine kinases)/MEK/ERK signaling pathway overactivation. This is consistent with upregulation and activation of Pyk2 in the pathogenesis of LVremodeling and HF^41–43^ and corroborates that in vitro Orai1 silencing or inhibition has been associated with a reduction of ERK1/2 and Pyk2 activity after phenylephrine treatment in neonatal cardiomyocytes^4^ and in glioma cells.^44^ In addition, cumulative evidence suggests that Pyk2 and ERK1/2 negatively regulate SERCA2a expression in neonatal, HL-1, and adult cardiomyocytes,^21,23,45–47^ and arterial smooth muscle.^22^ Our in vivo and in vitro results suggest that after hypertrophic insults, Orai1 inhibition is associated with blunted Pyk2 activation, which reduces SERCA2a downregulation, possibly in combination with MEK/ERK pathway.

Cardiac fibrosis remodeling is a major characteristic of pathological cardiac hypertrophy leading to a decrease in myocardial compliance and changes in electrical conduction.^48^ We showed that C-dnO1 mice after hypertrophic stress exhibited less interstitial fibrosis deposition and less type III collagen expression than WT mice. The cardioprotective effect of Orai1 inhibition is associated with restored Ca^2+^ homeostasis after cardiac insult but seems to also depend on fibrosis. Accordingly, some studies had proved a link between Orai1 and fibrosis.^10,49,50^ More important, it has been demonstrated that human ventricular fibroblasts from HF patients had increased collagen secretion capacity, which was related to increase in expression of Orai1 and SOCE.^50^

To explore the potential therapeutic relevance of the approach, Orai1 small-molecule inhibition was applied after hypertrophic insult and the development of left ventricular systolic dysfunction. In keeping with the results obtained in C-dnO1 mice, this translational approach showed that the progressive decline in ventricular function in vehicle-treated mice was prevented with JPIII treatment, without affecting cardiac hypertrophy. No fibrosis was observed when Orai1 is inhibited pharmacologically. Thus, we speculate an effect of JPIII in additional cell types in the heart, such as cardiac fibroblasts.

At a cellular level, the protective effect of JPIII as for C-dnO1 mice was associated with maintained SR Ca^2+^ load, normal SERCA2a expression and normal Pyk2/MEK/ERK1/2 activity.

These data suggest that targeted inhibition of Orai1 channels might be a promising strategy for maintaining contractility reserve after TAC. Although we did not observe immune compromise in our CD-1 toxicity study, given the important functional role of Orai1 in the immune system, it is an important consideration for this therapeutic approach moving forward.

It should be recognized that biochemical approaches such as western-blot and immunofluorescence have limited capabilities for proving the expression of a low abundance membrane protein such as Orai1 in a specific cell type such as cardiac myocytes. The antibody quality needs to be extremely high, but this has proven difficult to achieve for Orai1 through commercial suppliers or efforts of independent scientific groups such as ours. After extensive searching and validation, we have been able to provide evidence for cardiac myocyte Orai1 using these approaches, but we caution against conclusions based only on these data. Mass spectrometry capabilities have improved in recent years and could, in principle, provide complementary evidence. However, there are problems detecting natively-expressed low-abundance target proteins if they are difficult to solubilize and purify from complex samples; as is the case with membrane proteins such as Orai1 in cardiac myocytes. We performed mass spectrometry analysis after immunoprecipitation of Orai1 but could not detect Orai1 peptide even from whole heart, despite other expected peptides being readily resolved (unpublished data). Importantly, therefore, we took advantage of additional independent methods. A Mn^2+^ flux technique was applied to isolated cardiac myocytes, enabling us to demonstrate the presence of function Orai1 protein both using our newly-developed specific blocker of Orai1 (JPIII) and cardiac myocyte-specific expression of mutant dominant-negative Orai1 developed (C-dnO1). We went on to use C-dnO1 for extensive in vivo studies. These approaches have enabled exciting privileged insight, clarifying the importance of Orai1 in myocytes and potentially motivating important new therapeutic strategies for problems of HF, which we suggest should seek partial inhibition, not ablation, of Orai1.

Collectively, our findings underscore the concept that Orai1 might be a stress response molecule that is upregulated in cardiac hypertrophy, associated with Pyk2 and/or MEK/ERK cascade overactivation and SERCA2a downregulation, leading to depressed Ca^2+^ cycling and ventricular dysfunction as part of a maladaptive response.

**Table 7.**
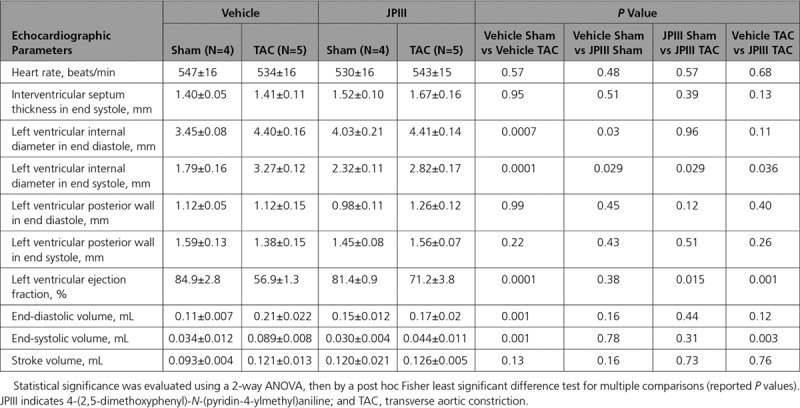
Cardiac Function Analyzed by Echocardiography 8 Weeks After TAC in Vehicle and JPIII-Treated Mice

## Acknowledgments

The authors thank the AnimEX plateform (Julie Burlot, Valérie Domergue) from the University of Paris-Sud, University of Paris-Saclay for animal care; Willian Mansfield, MRC Harwell, Cambridge, United Kingdom, for performing pronuclear injections to generate the C-dnO1 mice; Pfizer for the gift of PF-04520440; Bertrand Crozatier for advice on echocardiographic data; the Hospital Marie Lannelongue Microscopy Facility-Inserm UMR-S999, Le Plessis Robinson from the University of Paris-Saclay for confocal image acquisition.

## Sources of Funding

This work was supported by research grants from Inserm, the National Funding Agency for Research (ANR) (ANR-13-BSV1-0023, ANR-15-CE14–0005 and ANR-18-CE14-0023), Groupe de Réflexion sur la Recherche Cardiovasculaire et la Société Française de Cardiologie, CORDDIM (Cardiovasculaire Obésité Rein Diabète Domaine d’Intérêt Majeur, Région Ile de France), The Medical Research Council (G1002076), The British Heart Foundation (FS/18/12/33270, FS/13/23/30122, FS/12/54/29671, FS/17/2/32559, RG/17/11/33042, RG/15/7/31521) and a Cancer Research UK Fellowship.

## Disclosures

None.

## Supplementary Material
