# Supplementary material for: Orai1 Channel Inhibition Preserves Left Ventricular Systolic Function and Normal Ca2+ Handling After Pressure Overload
Source: Circulation. 2020 Jan 7;141(3):199–216. doi: 10.1161/CIRCULATIONAHA.118.038891 (PMC6970549; doi:10.1161/CIRCULATIONAHA.118.038891)
Supplement: Supplementary file 1 [file cir-141-199-s001.pdf]

SUPPLEMENTAL MATERIAL

Orai1 channel inhibition preserves left ventricular systolic function and normal Ca^{2+} handling after pressure overload

Running Title: Orai1 Ca^{2+} channels and ventricular function

Fiona Bartoli[1], PhD, Marc A. Bailey[2†], PhD MBChB MRCS, Baptiste Rode[2†], PhD, Philippe Mateo[1], PhD, Fabrice Antigny[3], PhD, Kaveen Bedouet[1], BS, Pascale Gerbaud[1], MSc, Rajendra Gosain[4], PhD, Jeffrey Plante[4], PhD, Katherine Norman[4], MChem, Susana Gomez[1], MSc, Florence Lefebvre[1], MSc, Catherine Rucker-Martin[3], PhD, Justin F.X. Ainscough[2], PhD, Mark T. Kearney[2], MD MBChB FRCP, Alexander-Francisco Bruns[2], PhD, Jian Shi[2], PhD, Hollie L. Appleby[2], PhD, Richard S. Young[2], PhD MBChB FRCS, Heba M. Shawer[2], MRes, Marjolaine Debant[2], PhD, Ana-Maria Gomez[1], PhD, David J. Beech[2], PhD FMedSci, Richard Foster[3], PhD, Jean-Pierre Benitah[1], PhD, Jessica Sabourin[1], PhD

Supplemental Methods

Chemicals

Nifedipine and caffeine were obtained from Sigma-Aldrich. KB-R7943 was obtained from TOCRIS. Fluo-4/AM and Fura-2/AM were obtained from Thermo Fisher Scientific. PF-04520440 was a gift from Pfizer.

Generation of cardiac-specific dn-Orai1^{R91W} transgenic mice (C-dnO1)

Human Orai1 cDNA with the R91W dominant-negative mutation (dn-Orai1^{R91W}) was cloned into the pTRE vector from Clontech. After AseI restriction digestion, the transgene was purified and microinjected into the pronuclei of (CBAxC57BL/6)F1 x (CBAxC57BL/6)F1 eggs[1, 2]. To identify transgenic founder mice, DNA from 21 day-old pups was analyzed for LacZ by PCR using primers LZ4 (5'-AATGGTCTGCTGCTGCTGAACG-3') and LZ5 (5'-GGCTTCATCCACCACATACAGG-3'). Each founder line was backcrossed 2 to 4 times on the C57BL/6 genetic background. The dn-Orai1^{R91W} transgene and LacZ reporter were under control of the tetracycline response element (TRE) and the CMV minimal promoter. Double-transgenics were generated by breeding with mice carrying transactivator transgene encoding: α-MHC.tTA[3] that drives hOrai1^{R91W} expression in the myocardium. Only double-transgenic animals expressed the functional dn-Orai1^{R91W} and LacZ construct. Single- and non-transgenic littermates were used as WT. All animals were maintained in GM500 individually ventilated cages (Techniplast) at 21°C 50–70% humidity, light/dark cycle 12/12 h on A03 diet (Safe) *ad libitum*. Used Primers for hOrai1 (5'-GCCAGAGTTACTCCGAGGTG-3'; 5' GCTGATCATGAGCGCAAACAG-3') and for α-MHC.tTA (5'-TTTCGATCTGGACATGTTGGG-3'; 5'-GGCATCGGTAAACATCTGCTC-3').

Ventricular myocytes isolation

Adult mouse ventricular cardiomyocytes were isolated using an enzymatic perfusion method as previously described [4].

Primary cell cultures of neonatal rat ventricular cardiomyocytes

Neonatal rat ventricular cardiomyocytes (NRVMs) were isolated using enzymatic digestion method as previously described[5]. 24 h after plating, NRVMs were stimulated with 100 µM phenylephrine for up to 48 h in presence or absence of 5 µM JPIII or 5 µM PF-04520440 during the final 24 h.

Cell Culture

WT HEK293 cells (CRL-1573™) were purchased from the American Type Culture Collection (ATCC) and grown in DMEM supplemented with 10% FBS and 100 units/mL penicillin/streptomycin. HEK293 cells stably over-expressing exogenous murine TRPC6 or stably over-expressing tetracycline regulated human TRPC5-TRPC1, TRPC4β-TRPC1, TRPC5 or TRPM2 have been previously published by our group[6-10]. To generate HEK-Orai3 cells under a tetracycline inducible promotor, T-REx™-293 cells were transfected with pcDNA4/TO-Orai3 using Lipofectamine 2000 (Thermo Fisher Scientific) for 24 h. Subsequently, cells were treated with blasticidin and zeocin to select stably transfected cells. For the tetracycline regulated systems, transgene expression was induced with 1 µg/mL tetracycline (or 10 ng/mL tetracycline for the HEK-Orai3 cells) for 24 h prior to experiments. mCherry-Orai1 in a p3XFLAG7.1 backbone was a kind gift from Schmuel Muallem, NIH. mCherry-Orai1-SS was designed as per Li and colleagues[11] by inserting 2 tandem S domains of STIM1 (residues 336–485) to the C-terminal end of Orai1 separated by 2 linker sequences, L1: GGSGGSGGGILQSRGGSGGSGGSG and L2: TSRGYQATMGGSG (mCherry-Orai1-

SS). Synthesis of the clone was performed by GeneArt gene synthesis, Thermo Fisher Scientific. mCherry-Orai1-SS was transfected into HEK293 cells using Lipofectamine 2000 for 24 h prior to experiments. mCherry-Orai1-SS transfected cells were maintained in 10 µM $LaCl_3$ prior to experiments and transfection efficiency evaluated using an IncuCyteZOOM (Essen Bioscience) fluorescent microscope housed within a standard tissue culture incubator. All cells were maintained at 37°C in a 5% CO_2 incubator and tested negative for mycoplasma contamination.

Orai1 knockdown in pulmonary arterial smooth muscle cells

PASMCs (Pulmonary arterial smooth muscle cells) were isolated from pulmonary arteries obtained during lobectomy or pneumonectomy for localized lung cancer from control subjects. Pulmonary arteries were studied at a distance from tumor areas. Patients studied were part of a program approved by our institutional Ethics Committee, and had given written informed consent (ID RCB: 2018-A01252-53, approved on June 18, 2006). PASMCs were then transfected in suspension by incubating 4×10^5 cells in a solution containing 500 µL of Opti-MEM, 3 µL of Lipofectamine RNAiMax and 100 nM of a specific *Silencer*™ Select siRNA against Orai1 (sense siRNA sequence: GCCUGAUCUUUAUCGUCUUtt; antisense siRNA sequence: AAGACGAUAAAGAUCAGGCcg) or *Silencer*™ Select negative control (Si scramble) according to manufacturer protocols (Thermofisher Scientific).

Generation of Orai1 deficient fibroblasts

cKO Orai1 mice (hereafter referred to as floxed Orai1 mice) in which exons 2 and 3 of Orai1 are flanked with LoxP sites as described in[12] were originally produced by Amgen and kindly provided by Stefan Feske (New York University, USA). Primary murine fibroblasts were isolated from ear-notches of homozygous $Orai1^{fl/fl}$, heterozygous $Orai1^{fl/+}$ and WT $Orai1^{+/+}$

mice, via enzymatic digestion using an enzyme solution of type II collagenase (1 mg/mL) in DMEM. To achieve *in vitro* Cre recombinase-mediated Orai1 deletion, cells at passage 1 were treated overnight with 8 μM of cell-permeable TAT-Cre recombinase (Millipore, SCR508) diluted in DMEM. Experiments were performed using cells at passages 2–4. The presence of loxP site was verified by PCR analysis of DNA isolated from the fibroblast cells. PCR was carried out using Q5 high fidelity enzyme (NEBL, E0555S), and primers spanning the 5' loxP site of the floxed Orai1 allele (5'-CAGCGTGCATAATATACCTAACTCTACCCG-3', 5'-GTATTGATGAGGAGAGCAAGCGTGAATC-3').

Blue-gal staining

Adult mouse ventricular cardiomyocytes or heart sections were washed twice with ice-cold PBS and fixed for 15 min in 2% formaldehyde, 0.8% glutaraldehyde, 0.02% igepal NP-40, 1 mM $MgCl_2$, 0.1 mg/mL Na-deoxycholate in PBS, at 4°C. Cells were then washed twice with ice-cold PBS 1X and incubated for at least 2 h at room temperature in blue-gal staining solution containing 0.4 mg/mL Bluo-gal (Sigma-Aldrich), 5 mM K-ferrocyanide, 5 mM K-ferricyanide, 1 mM $MgCl_2$ in PBS. After staining, cells were washed in PBS and imaged with Nikon Diaphot 300 inverted microscope equipped with Nikon D300S camera. The blue-gal staining was done after each isolation of ventricular cardiomyocytes validating LacZ and dn-Orai1^{R91W} expression.

Quantitative Real-Time PCR

The RNA extraction and RT-qPCR quantification were performed as previously described [5]. The primers used in this study are listed in Table S1.

Table S1: List of primers used for RT-qPCR

Gene	Forward (5'-3')	Reverse (5'-3')
hOrai1	TGG CAA TGG TGG AGG TG	CTG CTT CTT GAG GGG C
mOrai1	ATC GTC TTT GCC GTT CAC TT	AGA GAA TGG TCC CCT CTG TG
Nppa	AGG CCA TAT TGG AGC AAA TC	CTC CTC CAG GTG GTC TAG CA
Nppb	ATG GAT CTC CTG AAG GTG CTG	GTG CTG CCT TGA GAC CGA A
Acta1	CGT GAA GCC TCA CTT CCT ACC	AGA GCC GTT GTC ACA CAC AA
TRPC6	TGG CAC ACA ACA AAC AAT CA	ATC AAT CTG GGC CTG CAA TA
RPL32	GCT GCT GAT GTG CAA CAA A	GGG ATT GGT GAC TCT GAT GG
YWHAZ	AGA CGG AAG GTG CTG AGA AA	GAA GCA TTG GGG ATC AAG AA
TBP	AAA GAC CAT TGC ACT TCG TG	GCT CCT GTG CAC ACC ATT TT
SERCA2a	CAC ACC GCT GAA TCT GAC	GGA AGC GGT TAC TCC AGT
STIM2	GCT AAG GAC GAG GCA GAA AA	GGT CCC AAA GAC TGT GCT TC
Orai3	CCA CCA GTC ACC ACA CCA	CCA GCC CAC CAG AAC AAC

Western-blot

Western-blot was performed as described previously[5] and the used antibodies are listed in Table S2. Of note, two ultra-sensitive enhanced chemiluminescent (ECL) substrates for low femtogram level detection and for pictogram amounts of target protein were used. Different protein ladder were used (Prestained Protein Ladder from Euromedex (Lot 272004/15_47), PageRuler Plus Prestained Protein Ladder from Thermofischer and Precision Plus Protein™ Kaleidoscope™ Prestained Protein Standards from Biorad). As a negative control, the Orai1 antibody was neutralized overnight at 4°C with 9 times excess blocking peptide synthesised by Eurogentec (H-HRG DHP LTP GSH YA-OH) that corresponds to the epitope recognized by the Orai1 antibody from Sigma-Aldrich.

Table S2: List of primary antibodies used for Western-blot

Protein	Host	Dilution	Code	Source
TRPC1	mouse	1/200	SC-133076	Santa Cruz
TRPC3	rabbit	1/200	ACC-016	Alomone
TRPC4	rabbit	1/200	ACC-018	Alomone
TRPC5	mouse	1/200	73-104	UC Davis/NIH NeuroMab Facility
TRPC6	rabbit	1/200	ACC-017	Alomone
Orai1	rabbit	1/200	O8264	Sigma
Orai3	rabbit	1/200	ACC-065	Alomone
STIM1	rabbit	1/200	S6197	Sigma
STIM2	rabbit	1/200	ACC-064	Alomone
SERCA2a	mouse	1/500	SC-376235	Santa Cruz
PLB total	rabbit	1/5000	8495S	Cell Signaling
PLB P-Ser16	rabbit	1/5000	A010-12	Badrilla
PLB P-Thr17	rabbit	1/10000	A010-13	Badrilla
RyR2 total	mouse	1/1000	MA3-925	ThermoFisher
RyR2 P-Ser2808	rabbit	1/2000	A010-30AP	Badrilla
RyR2 P-Ser2814	rabbit	1/2000	A010-31AP	Badrilla
CaMKII P-Thr286	rabbit	1/200	SC-32289	Santa Cruz
CaMKII Total	rabbit	1/200	SC-517278/100362	Santa Cruz
Pyk2 P-Tyr402	rabbit	1/200	#3291	Cell Signaling
Pyk2 total	rabbit	1/200	#3292	Cell Signaling
MEK1/2 P-Ser217/221	rabbit	1/1000	#9121	Cell Signaling
MEK1/2 Total	rabbit	1/1000	#9122	Cell Signaling
ERK1/2 P-Thr202/Tyr204	rabbit	1/1000	#9101	Cell Signaling

ERK1/2 Total	rabbit	1/1000	#9102	Cell Signaling
β-actin HRP	mouse	1/30000	SC-47778	Santa Cruz

Immunostaining

Ventricular cardiomyocytes were fixed and permeabilized in cold 100% methanol or with 2% paraformaldehyde (PFA)/0.5% Triton X-100 as previously described[13]. Co-immunostaining of Orai1 with WGA (Wheat Germ Agglutinin, Alexa Fluor™ 488 Conjugate; Thermofisher Scientific; W11261; 1/100) or with α-actinin (Sigma-Aldrich; A7811; 1/800) was also performed. As a negative control, the Orai1 antibody was neutralized 1 h at room temperature with 9 times excess blocking peptide synthesised by Eurogentec (H-HRG DHP LTP GSH YA-OH) that corresponds to the epitope recognized by the Orai1 antibody from Sigma. We also performed negative control without the primary antibody.

Immunohistochemistry of heart sections

Mice hearts were fixed in 4% PFA for 2 h at 4°C and embedded in 4% agarose low gelling temperature. Hearts were sliced in 80 µm sections using a Vibratome (Leica Microsystems) and permeabilized in PBS/Triton X-100 0.5% for 1 h at room temperature. Sections were blocked in PBS/Triton X-100 0.1%/3% BSA for 2 h at room temperature and incubated overnight at 4°C with anti-Orai1 polyclonal antibody (Sigma-Aldrich (O8264) at 50 µg/mL). Next, sections were incubated in PBS/Triton X-100 0.1%/3% BSA overnight at 4°C with secondary goat anti-rabbit antibodies coupled to AlexaFluor™ 488 or 555. Finally, sections were mounted with Prolong gold antifade including DAPI to counterstained nuclei and imaged with a confocal microscope (LSM700 (Carl Zeiss)). As negative controls, the Orai1 antibody was neutralized overnight at room temperature with 9 times excess the blocking peptide or the heart sections were incubated without the primary antibody.

Histological analysis of cardiac fibrosis

Hearts were fixed in 4% PFA, embedded in paraffin and serially sectioned (5 µm). Sections were stained with Picrosirius Red F3BA then observed with an Axio Observer Z1 (Carl Zeiss). Pictures were recorded at x20 and interstitial fibrosis was quantified on 2 sections (20-25 fields/section) per animal, with ImageJ software, as previously described[14].

Measurement of cation influx using Fura-2 fluorescence quenching by $MnCl_2$

Freshly isolated ventricular cardiomyocytes were incubated for 15 min with 1 µM Fura-2/AM solved in DMSO plus 20% pluronic acid. The Mn^{2+} influx was measured as described previously[5]. Ventricular cardiomyocytes were depleted with 5 µM thapsigargin and 10 mM caffeine in a Ca^{2+}-free solution containing 140 mM NaCl; 4 mM KCl, 1.1 mM $MgCl_2$, 10 mM HEPES, 0.1 mM EGTA, 10 µM nifedipine and 5µM KB-R7943, inhibitors of L-type Ca^{2+} channels and NCX exchanger, respectively. Then, a 500 µM $MnCl_2$ solution was perfused in presence of nifedipine and KB-R7943 that induces quenching of Fura-2 fluorescence and the initial and linear slope of the Fura-2 fluorescence's decrease was measured reflecting the SOC channel activity.

Ca^{2+} imaging

The $[Ca^{2+}]_i$ transients were measured as described previously[15]. Freshly isolated ventricular cardiomyocytes were incubated for 30 min at room temperature with 7 µM of Fluo-4/AM. The Fluo-4 Ca^{2+} signal was recorded with a laser scanning confocal microscope (Leica SP5) equipped with a ×40 water immersion objective. Fluo-4 was excited at 490 nm with a white light laser, and emission was collected at >510 nm. The cardiomyocytes, continuously perfused with physiological saline solution, were paced at 1 Hz. $[Ca^{2+}]_i$ transients were

recorded by scanning the cells in XT line mode. The cytosolic Ca^{2+} variation was normalized by dividing the peak fluorescence intensity (F) by the fluorescence intensity at rest (F_0) to generate an F/F_0 image after background subtraction. $[Ca^{2+}]_i$ transient properties were evaluated from the F/F_0 fluorescence trace: peak F/F_0 and the time constant of decay (ms), which was calculated by fitting the decay portion of the fluorescence trace to a monoexponential function. For SR Ca^{2+} load estimation, cardiomyocytes were rapidly perfused with 10 mM caffeine just after field stimulation. The amplitude of caffeine-evoked $[Ca^{2+}]_i$ transients was used to assess SR Ca^{2+} load. Analysis was performed using IDL imaging software.

High throughput Ca^{2+} imaging

Cells were plated at 90-100% confluence in poly-d-lysine coated black/clear 96 well microplates. On the day of the experiment, cells were loaded with 2 µM Fura-2/AM (Molecular Probes) with 0.01% pluronic acid in standard bath solution (SBS) for 1 h at 37°C followed by a wash at room temperature for 30 min (including pre-treatment with inhibitors under test). SBS contained: 130 mM NaCl, 5 mM KCl, 8 mM D-glucose, 10 mM HEPES and 1.2 mM $MgCl_2$ and 1.5 mM $CaCl_2$ titrated to pH 7.4. To deplete cellular Ca^{2+} stores, cells were incubated in Ca^{2+} free SBS (130 mM NaCl, 5 mM KCl, 8 mM D-glucose, 10 mM HEPES, 1.2 mM $MgCl_2$ and 0.4 mM EGTA titrated to pH 7.4) and 1 µM thapsigargin (Sigma-Aldrich, T9033). Ca^{2+} flux was performed on a FlexStation (Molecular Devices) bench-top scanning fluorometer at room temperature (21-23°C). Ca^{2+} entry is presented as the change in intracellular calcium concentration ($Ca^{2+}{}_i$) as measured by the ratio of Fura-2 emission (510 nm) intensities for 340 nm:380 nm excitation (ΔF Ratio). The SOFTMax® Pro software v4.3.1 (Molecular Devices) was used for data collection. In the case of store depletion experiments, extracellular Ca^{2+} was added back at 0.3 mM to observe SOCE.

Otherwise a range of agonists were added to stimulate Ca^{2+} entry in the continued presence of 1.5 mM Ca^{2+}: 5 µM (Orai1 stimulation) or 75 µM (Orai3 stimulation) 2-aminoethoxydiphenyl borate (2-APB), 20 µM gadolinium (Gd^{3+}), 100 µM carbachol, 100 nM (-)-Englerin A ((-)-EA), 1 mM H_2O_2.

Synta 66 (S66, GSK1349571A; N-(2',5'-Dimethoxy[1,1'-biphenyl]-4-yl)-3-fluoro-4-pyridinecarboxamide) and novel compounds were synthesised in-house (by K.N., R.G., & R.F.) and purity confirmed by NMR spectroscopy and LC-MS. A detailed synthetic scheme for JPIII and the internal standard, $JPIII_{D6}$ is included in the supplementary methods for chemical synthesis section. Orai1 inhibitors were either tested at a single concentration (5 or 10 µM) or at a range of concentrations from 0.001-10 µM to allow construction of dose response curves. The IC_{50} was determined by fitting a Hill equation to the dose response data.

Action potential recording

Action potentials (AP) were recorded using whole-cell patch-clamp method with solutions and protocols as previously described[16].

Patch-clamp recording

Whole-cell patch-clamp recordings were achieved with Axopatch-200B (Axon Instruments, Inc.) equipped with Digidata 1550B and pCLAMP 10.6 software (Molecular Devices, Sunnyvale, CA, USA) at room temperature. The recording glass pipettes had tip resistance between 3 and 5 MΩ. The currents were sampled at 20 kHz and filtered at 2 kHz. The external solutions consisted of (in mM) 140 NaCl, 4 CsCl, 10 $CaCl_2$, 2 $MgCl_2$ and 10 Na-HEPES (pH 7.4 with NaOH). The pipette solution contained (in mM) 130 caesium glutamate, 4 $CaCl_2$, 1 MgATP, 5 $MgCl_2$, 10 EGTA and 10 Na-HEPES (pH 7.2 with NaOH). After HEK293T cells overexpressing Orai1 and STIM1 were pre-treated with 1 µM thapsigargin, a

100 ms voltage ramp protocol from -100 mV to 100 mV was applied to observe CRAC channel currents. Then, various concentrations of JPIII were superfused. The inhibitory effect of JPIII was calculated with the current values at -100 mV.

Telemetric ECG studies

ECG was recorded in ambulatory mice using implantable ECG telemeters (Data Sciences International). ECG telemeters were sterilized and store in sterile saline solution before the implantation. The mice were anesthetized using 2% isoflurane in 0.8 L/min 100% O_2. A vertical midline incision was created in the skin overlying the back. The skin was carefully separated from underlying connectives tissues using blunt-ended scissors. The telemeter was inserted into the cavity and additionally, a small hole was created in the mouse's right upper chest skin and another in the left abdomen skin. Both leads were pull up through the lead hole using forceps to obtained an implantation in « lead two » configuration with the white (negative) lead implanted in the upper chest and the red (positive) lead in the left abdomen. Negative and positive leads were respectively anchored to the pectoral muscle and to the underlying peritoneal tissue using 6.0 Prolene suture. The skin incisions were closed with continuous suture. ECG recording was performed 7 days after the surgery using RPC-1 small animal receiver (DSI) during 24 h.

Transverse Aortic Constriction

Cardiac hypertrophy was induced by transverse aortic constriction (TAC) under anesthesia (intraperitoneal injection of ketamine 50 mg/kg and xylazine 8 mg/kg) in male mice (8 weeks of age). After thoracotomy, the aortic arch was constricted between the brachiocephalic and the left common carotid arteries with a thread (4.0 Prolene suture) around a blunt 26-gauge needle (Ø 0.42 mm). The knots were tied against the needle before removing it, leaving a

region of stenosis which partially reduced the vascular diameter. Sham-operated mice underwent the same procedure without aortic ligation and served as controls. Mice were left for 5 weeks to develop cardiac hypertrophy and using M-mode echocardiography, cardiac contractile function was assessed before and after the surgery.

Echocardiography and Doppler

Transthoracic echocardiography was performed using an echocardiograph (Vivid 9, GE Healthcare) equipped with a 15-MHz linear transducer, under 2% isoflurane gas anesthesia in 0.8 L/min 100% O_2. The thickness of the left ventricular anterior and posterior walls were measured in short and long axis at papillary muscle level using two-dimensional-guided (2D) M-mode echocardiography during systole and diastole, and contractile parameters such as fractional shortening (FS %) or ejection fraction (EF %) were calculated from these measurements. For anatomical parameter, the whole heart weight is always normalized to body weight or tibia length. The left ventricular mass is an echocardiography-based left ventricular mass estimation calculated by a most commonly used Penn formula for rodents: LV mass (Penn) = 1.04 ($[LVIDd + LVPWd + IVSd]^3$- $[LVIDd]^3$).[17]

Echocardiographic examinations were performed in a blinded fashion.

Tail cuff plethysmography

Blood pressure was measured in awake, restrained mice maintained at 37°C in a heated chamber, using a CODA™ non-invasive tail cuff plethysmography system (Kent Scientific). Mice received one training session the day prior to recording. In each experimental run, each mouse had 10 acclimatisation cycles followed by 18 data recording cycles with a 5 second rest between each cycle.

LC-MS for JPIII

Blood was sampled from the inferior vena cava for terminal samples and the saphenous vein for samples in awake animals on clear plastic and allowed to clot before centrifugation at 13 000 RPM for 20 min to obtain serum and stored at -80°C until analysis. JPIII was detected by LC-MS on a Bruker Daltronics using a gradient of increasing acetonitrile (0-100%) in 0.1% trifluoroacetic acid at 1 mL/min after passage of samples through a Pierce™ ^{18}C reversed-phase resin spin column with centrifugation at 13 000 RPM and elution in 0.05% trifluoroacetic acid in acetonitrile. To quantify JPIII in samples, 200 ng/mL of a deuterated JPIII internal standard ($JPIII_{D6}$) was added to the sample and compared with the unknown quantity of JPIII to yield a JPIII concentration.

In vitro ADMET

Aqueous solubility was assessed at pH 7.4 by dissolving inhibitors in DMSO then diluting in PBS to a final concentration of 1-100 µM, incubating for 2 h and measuring absorbance at 620 nm (Cyprotex). For JPIII, the broad solubility range derived from the colorimetric assay was validated with accuracy by thermodynamic stability at Cyprotex. Plasma stability was determined at 5 time points following the addition of 1 µM JPIII to murine plasma by LC-MS at Peakdale Molecular. The fraction of 5 µM compound unbound after exposure to 10% mouse plasma was assessed by equilibrium dialysis (Cyprotex). Metabolic stability was assessed in 0.5 mg/mL murine or human liver microsomes driven by NADPH. Compounds were tested at 3 µM at 0, 5, 15, 30 and 45 min to determine the intrinsic clearance (CL_{int}) and half-life ($t_{1/2}$, Cyprotex). Hepatic stability was assessed by exposing 3 µM of the test compound to murine hepatocytes. Samples were taken at 0, 5, 10, 20, 40 and 60 min and subjected to LC-MS to establish the CL_{int} and $t_{1/2}$ (Cyprotex). To determine unidirectional cell permeability, Caco-2 cells were seeded onto Milicell (Merk Milipore) plates and allowed to

form a confluent monolayer over 20 days. On day 20, the compound was added to the apical side of the monolayer and transport across the monolayer measured over 2 h at 37°C and permeability coefficient (P_{app}) derived (Cyprotex).

In vivo chronic treatment

Osmotic minipumps (model 1004, Alzet) were implanted subcutaneously via posterior neck incision 5 weeks after TAC or sham-surgery, leading to constant delivery of JPIII at 500ng/kg/min (0.72 mg/kg/day in DMSO) or vehicle only. Explantation of the osmotic pumps at the end of the experiment confirmed empty reservoirs and a lack of precipitation around the nozzle. M-mode echocardiography was performed in each animal before the implantation to evaluate the degree of cardiac hypertrophy induced by TAC and 3 weeks after the implantation of osmotic minipumps to evaluate cardiac function under JPIII treatment.

Chemical Synthesis

Instrumentation

Reagents and solvents were obtained from commercial supplier and used without further purification. Thin layer chromatography (TLC) analyses were conducted using silica gel (aluminium foil backing) plates and visualised under UV radiation in a dark-box. Compound purification was effected using gradient elution on a Biotage Isolera-4 running SiO_2 cartridges. HPLC-MS was performed on a Bruker Daltronics spectrometer running a gradient of increasing acetonitrile (0 to 100%) in water containing 0.1% TFA at 1 mL/min on a short path ^{18}C reverse phase column detecting compounds with both a diode array detector and a Bruker Mass spectrum analyser. HRMS experiments were conducted on a Bruker MaxisImpact time-of-flight spectrometer operating in a positive ion mode with sodium formate as an internal standard. ^{1}H, ^{13}C experiments were recorded using either a Bruker

DRX 500 instrument or a Bruker DPX 300 operating at 298K, by solubilising the sample in deuterated chloroform ($CDCl_3$) with internal standard tetramethylsilane (TMS), CD_3OD, or d_6-acetone as the NMR solvent. Chemical shifts were expressed as parts per million (ppm) and the splitting signals of NMR assigned as s (singlet), d (doublet), t (triplet), dd (doublet of doublet), br (broad) or m (multiplet).

Preparation of 4-(2,5-dimethoxyphenyl)-N-[(pyridin-4-yl)methyl]aniline (JPIII)

Synthetic route:

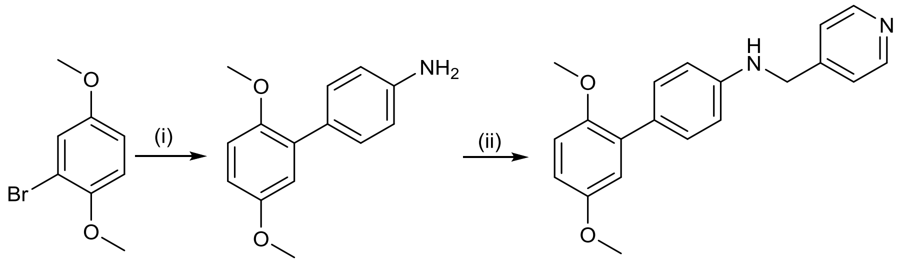

1 2 JPIII

(i) Pd(dppf)Cl_2-CH_2Cl_2, 4-aminophenylpinacolboronate, dioxane-H_2O, K_2CO_3, 110°C, 88%; (ii) methanol, $NaBH_4$, 4-pyridinecarboxaldehyde, 34%.

4-(2,5-dimethoxyphenyl)aniline (**2**)

A dioxane (8 mL) and water (2 mL) mixture comprising of 2,5-dimethoxybromobenzene (**1**) (260 mg, 1.23 mM), potassium carbonate (660 mg, 4.80 mM) and 4-aminophenylpinacolboronate ester (180 mg, 0.82 mM) was stirred for 20 min at room temperature, before Pd(dppf)Cl_2-CH_2Cl_2 (5 mg) was added and the contents degassed for 10 min, prior to the flask being lowered onto a heating block at 110°C. The reaction mixture was then heated under reflux with stirring for 4 h, by which time, TLC had indicated complete consumption of the precursors. The contents were cooled to room temperature, diluted with

aqueous saturated ammonium chloride (20 mL) and stirred for 10 min before being extracted with dichloromethane, which, was dried over sodium sulfate. Filtration and evaporation under reduced pressure afforded a dark residue which was purified by chromatography (SiO_2; ethyl acetate: hexane = 1:1) to afford the title compound (**2**) as a yellow oil that solidified upon standing (167 mg, 88%). HPLC-MS: 1.55 min, 230.1 $[M+H]^+$. ^1H NMR (500 MHz, $CDCl_3$): δ 7.39 (dd, *J* = 6.5 Hz, 2 Hz, 2H), 6.92 (m, 2H), 6.82 (dd, *J* = 9 Hz, 5 Hz, 1H), 6.76 (dd, *J* = 6.5 Hz, 2.5 Hz, 2H), 3.83 (s, 3H), 3.78 (s, 3H).

4-(2,5-dimethoxyphenyl)-N-[(pyridin-4-yl)methyl]aniline ***(JPIII):***

A methanol (3 mL) solution containing 4-(2,5-dimethoxyphenyl)aniline (**2**) (51 mg, 0.22 mM) and 4-pyridinecarboxaldehyde (27 mg, 0.25 mM) was heated under reflux with stirring for 3 h, before the contents were cooled to room temperature and chilled in an ice-water bath. Sodium borohydride (28 mg) was added in a single portion and the contents left to stir for 12 h at room temperature, before being quenched with several drops of aqueous ammonium chloride. The resulting suspension was sonicated and then stirred for 10 min before being extracted with dichloromethane and dried over sodium sulfate. Concentration under reduced pressure afforded the crude residue, which was purified *via* chromatography (SiO_2: ethyl acetate: hexane = 4: 1) to yield the title compound as a yellow waxy solid (26 mg, 34%). HPLC-MS: 1.62 min, 321.2 $[M+H]^+$. ^1H NMR (500 MHz, $CDCl_3$): δ 8.60 d, *J* = 5.5 Hz, 2H), 7.41 (d, *J* = 8.6 Hz, 2H), 7.35 (d, *J* = 5.6 Hz, 2H), 6.91 (m, 2H), 6.81 (dd, *J* = 8.8 Hz, 3.2 Hz, 1H), 6.64 (d, *J* = 8.7 Hz, 2H), 4.45 (s, 2H), 4.21 (br, 1H), 3.84 (s, 3H), 3.77 (s, 3H); ^{13}C NMR (125 MHz, $CDCl_3$): δ 153.8, 150.8, 150.01, 149.02, 146.6, 131.6, 130.4, 128.1, 122.1, 116.4, 112.6, 112.5, 112.2, 56.3, 55.8, 47.2. HRMS (*m/z*): $[M+H]^+$ calcd. for $C_{20}H_{20}N_2O_2$, 321.1598; found, 321.1595.

Preparation of D_6-4-(2,5-dimethoxyphenyl)-N-[(pyridin-4-yl)methyl]aniline ($JPIII_{D6}$)

Synthetic route:

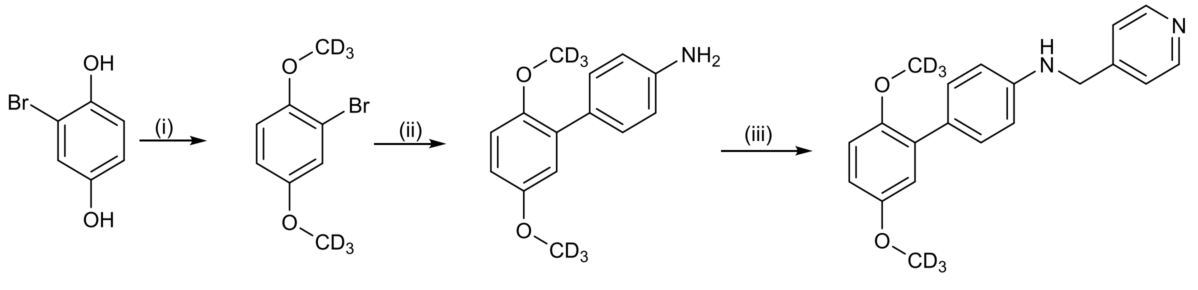

(i)I-CD_3, Cs_2CO_3, MeCN, rt; (ii) Pd(dppf)Cl_2-CH_2Cl_2, 4-aminophenylpinacolboronate, dioxane-H_2O, K_2CO_3, 100°C, (iii) methanol, $NaBH_4$, 4-pyridinecarboxaldehyde, 14% (over 3 steps).

An acetonitrile (2 mL) suspension of bromohydroquinone (**1**) (50 mg, 0.25 mM) was treated with cesium carbonate (215 mg, 0.66 mM) under nitrogen and stirred for 20 min at room temperature in the dark, before, d_3-iodomethane (100 mg, 0.67 mM) was added in a single portion via syringe and the mixture stirred for a further 18 h at room temperature. Following this, Amberlyst A21 basic resin (500 mg) was added and the contents slowly stirred for a further hour, and filtered under suction through celite. The resin cake was washed with TBDME and the combined organic portions were concentrated to afford the crude compound (**4**) as volatile pale yellow oil that was used directly in the next step. $JPIII_{D6}$ was prepared according to the method for the preparation of JPIII using deuterated aromatic bromide (**4**) (21 mg, 0.1 mM) and 4-aminophenylpinacolboronate ester (20 mg, 0.1 mM) in the presence of potassium carbonate (40 mg, 0.30 mM) and Pd(dppf)Cl_2-CH_2Cl_2 (1 mg) to afford the crude biphenyl aniline (**5**) as a beige powder which was used in the next step without purification (HPLC-MS: 1.51 min, 236.1 $[M+H]^+$). This material was then stirred with 4-pyridinecarboxaldehyde (12 mg, 0.11 mM) in the presence of sodium borohydride (8 mg, 0.21 mmol) using the method employed for synthesis of JPIII to generate the title compound

(**JPIII $_{\mathbf{D6}}$**) as a yellow solid (4 mg, 14% over 3 steps). HPLC-MS: 1.64 min, 327.4 $[M+H]^+$. ^{1}H NMR (500 MHz, $CDCl_3$): δ 8.54 d, *J* = 5.0 Hz, 2H), 7.51 (d, *J* = 6.0 Hz, 2H), 7.37 (d, *J* = 8.5 Hz, 2H), 6.88 (m, 2H), 6.80 (dd, *J* = 8.0 Hz, 3.0 Hz, 1H), 6.60 (d, *J* = 8.5 Hz, 2H), 4.52 (d, *J* = 5.5 Hz, 2H), 4.38 (br, 1H); ^{13}C NMR (125 MHz, MeOD): δ 153.9, 151.6, 150.7, 148.5, 147.0, 131.9, 129.3, 127.1, 122.5, 121.9, 115.7, 112.4, 111.9, 45.8. HRMS (*m/z*): $[M+Na]^+$ calcd. for $C_{20}H_{14}D_6N_2O_2$, 349.1794; found, 349.1792.

Toxicology

Outbred Swiss CD-1 (International Genetics Standard) mice were purchased from Charles River (UK). Male mice at 10 weeks of age were used for the toxicology experiment which was performed in accordance with AACC-DACC/ASVCP Joint Task Force Clinical Pathology Testing Recommendations for Non-Clinical Toxicity Safety Studies[18, 19]. Animals were randomly allocated to infusion of JPIII (500ng/kg/min) or vehicle via Alzet 1004 osmotic mini pump implanted subcutaneously. Animals were weighed weekly during the 28 day infusion period. At the end of the experiment, blood was collected from the inferior vena cava under terminal anaesthesia on EDTA before perfuse-fixation with 10 mL PBS followed by 5 mL of 4% PFA. Organs were weighed before 48 hour fixation in PFA, processing, embedding, sectioning and H&E staining. Haematology assessment was performed on a Siemens Advia 2120 full blood count analysis system in the Leeds Teaching Hospitals CPA Approved Pathology Laboratory, Department of Blood Sciences. All analysis was performed blinded to the treatment allocation of the animals.

Supplemental Figures and figure legends

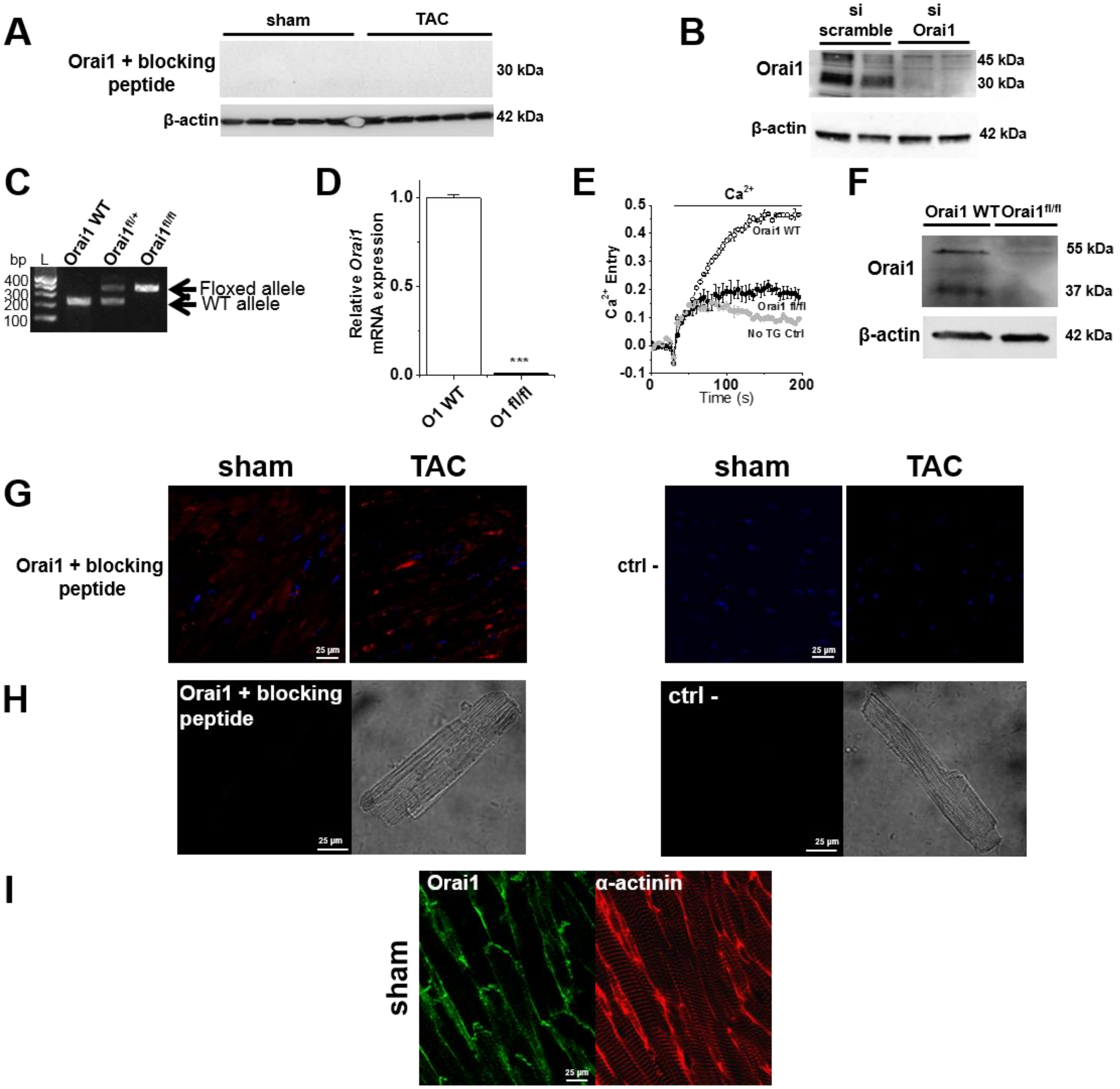

Supplemental Figure 1: Validation of the specificity of Orai1 antibody from Sigma-Aldrich (O8264) in ventricular tissues, in isolated ventricular cells, in pulmonary smooth muscle cells and in Orai1 knockout fibroblasts isolated from mice harbouring the floxed Orai1 allele (Orai1$^{fl/fl}$). **A,** Western-blot of ventricular tissue from sham and TAC mice with antigen pre-absorption with the corresponding blocking peptide. **B,** Western-blot with siRNA against Orai1 in pulmonary arterial smooth muscle cells from 2 healthy patients. **C,** Genotyping PCR of genomic DNA isolated from WT Orai1$^{+/+}$, heterozygous Orai1$^{fl/+}$ and homozygous Orai1$^{fl/fl}$ fibroblasts. The expected floxed Orai1 allele product size was 360 bp, and the WT allele product size was 220 bp using LoxP spanning primers. L, 100 bp DNA ladder; bp, base pairs.

D, Validation of Cre mediated excision by evaluation of mRNA expression of Orai1 in ear fibroblasts from Orai1 WT and Orai1$^{fl/fl}$ littermates exposed to *ex vivo* TAT-Cre. N=6 animals. ***P<0.001 *vs.* WT mice. **E,** Example FlexStation traces from the cells in (**D**) following store depletion with thapsigargin (Tg) and extracellular Ca^{2+} add back at 0.3 mM (N/n=5/45). Orai1 WT is shown as open symbols and Orai1$^{fl/fl}$ as closed black symbols. The no Tg control is shown in grey. **F,** Western-blot using the Orai1 antibody from Sigma-Aldrich (O8264) in the cells from (**D, E**). **G**, Negative controls were performed in ventricular sections. Left panel: with the blocking peptide against Orai1 antibody. Right panel: ctrl- corresponds to negative control without the primary antibody against Orai1. Scale bar, 25 µm. **H**, Negative controls were also performed in isolated cardiomyocytes. Scale bar, 25 µm. **I**, Representative immunohistochemistry with antibodies against Orai1 (green) and α-actinin (red) in sham mouse ventricular sections. Scale bar, 25 µm.

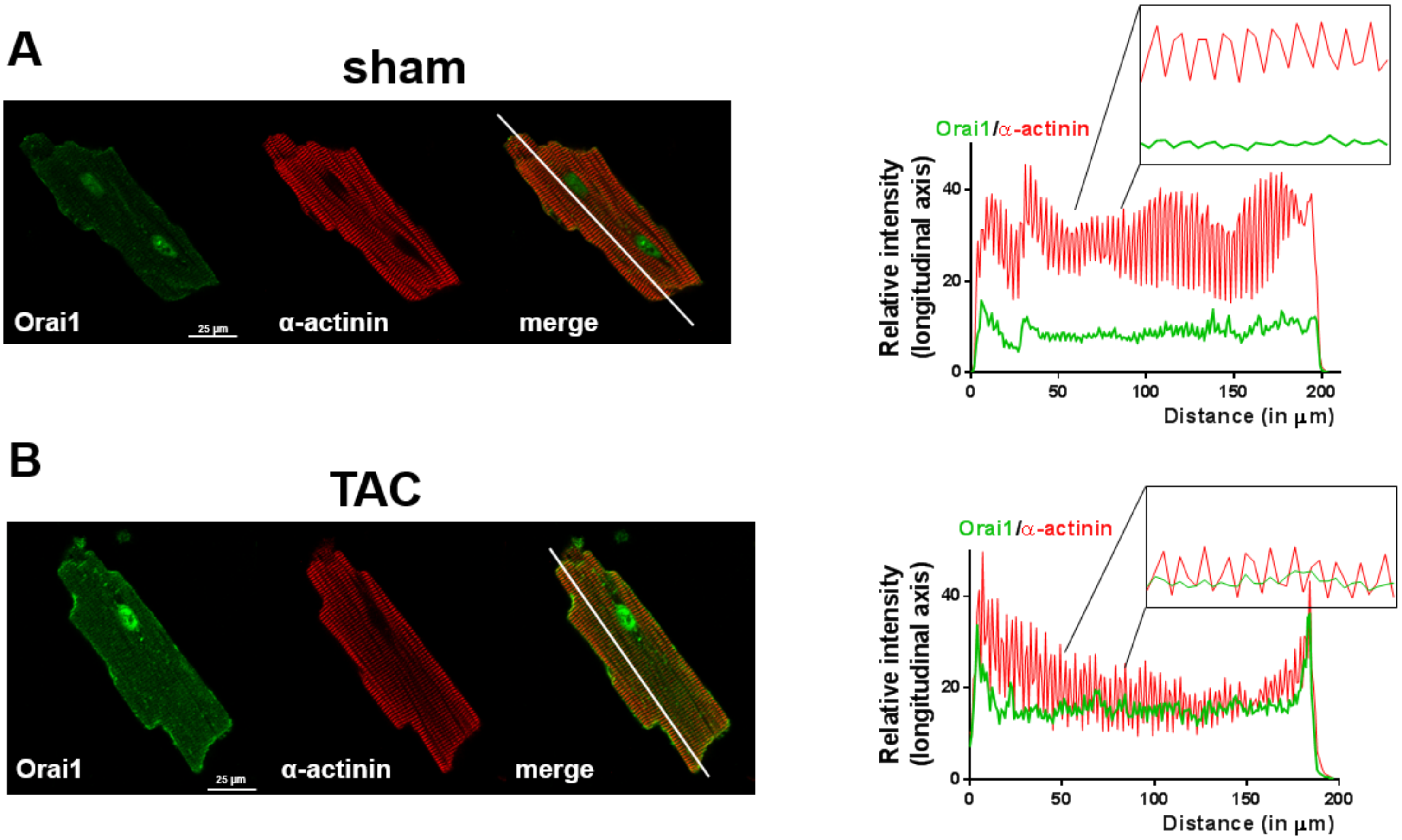

Supplemental Figure 2: Immunostaining of Orai1 in WT isolated ventricular cells. **A-B**, Immunostaining of Orai1 (green) and α-actinin (red) in ventricular cardiomyocytes from WT sham (**A**) or WT TAC (**B**) mice. White lines across ventricular cells (in longitudinal axis) show examples of regions from which pixel counts were taken. Scale bar, 25 µm.

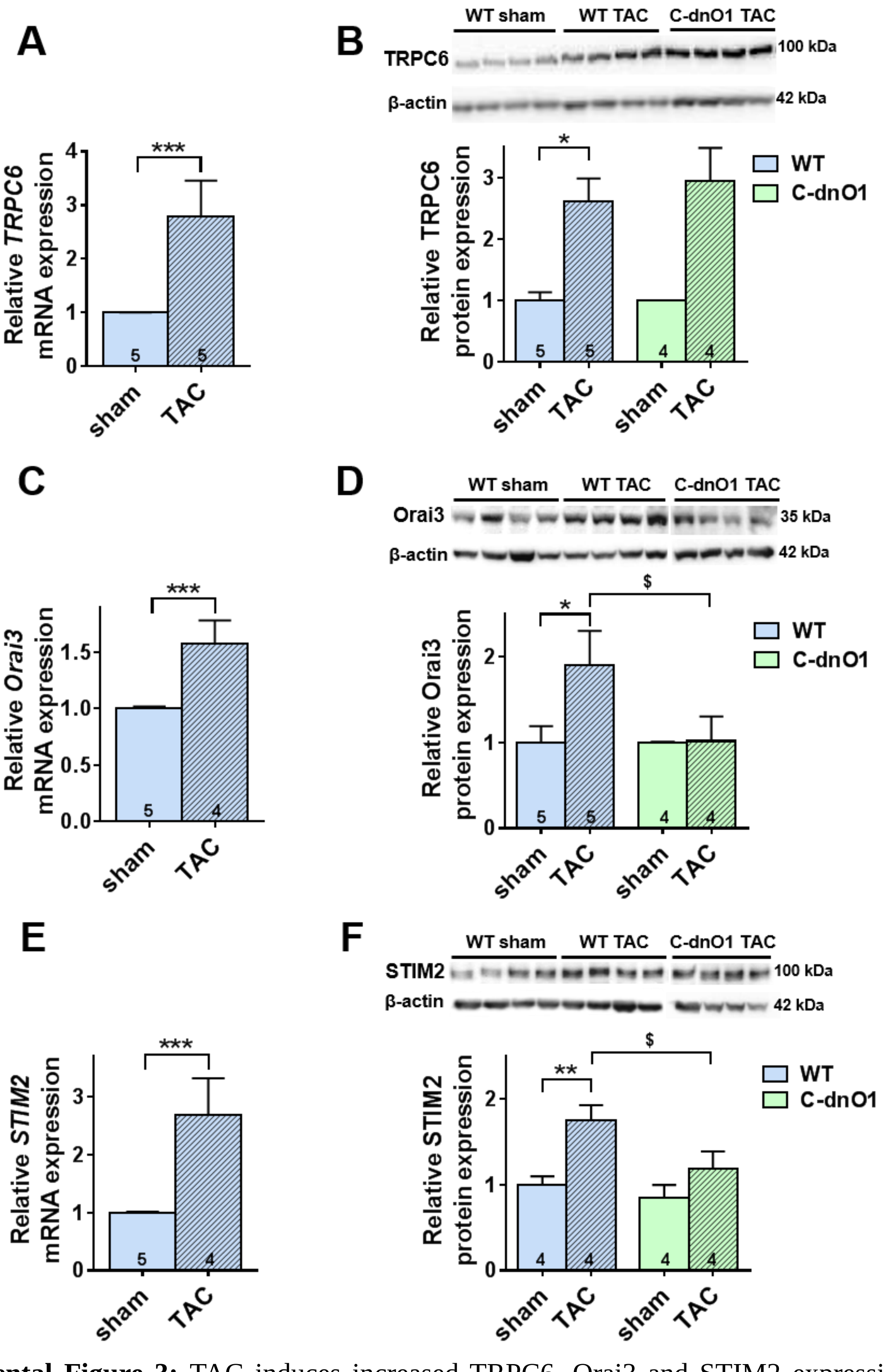

Supplemental Figure 3: TAC induces increased TRPC6, Orai3 and STIM2 expression in WT mice. **A**, **C**, **E**, Relative *TRPC6* (**A**), *Orai3* (**C**) and *STIM2* (**E**) mRNA expression was determined by RT-qPCR in ventricle tissue from WT sham or TAC mice after 5 weeks. mRNA levels were normalized to housekeeping genes and expressed as fold change of that determined in sham mice. N=4-5 animals. ****P*<0.001 *vs.* sham mice. **B**, **D**, **F**, Top panel: representative western-blot of TRPC6 (**B**), Orai3 (**D**) and STIM2 (**F**) in ventricle tissue from WT or C-dnO1 sham or TAC mice after 5 weeks. Bottom panel: relative TRPC6, Orai3 and STIM2 protein expression was evaluated by western-blot. Protein levels were normalized by

β-actin and expressed as fold change of that determined in sham mice. N=4-5 animals. $^{*}P<0.05$, $^{**}P<0.01$ *vs.* sham mice. $^{\$}P<0.05$ *vs.* WT TAC mice. Statistical significance was evaluated using Student's *t*-test (**A**, **C**, **E**) and two-way ANOVA followed by post-hoc Fisher LSD test for multiple comparisons (**B**, **D**, **F**).

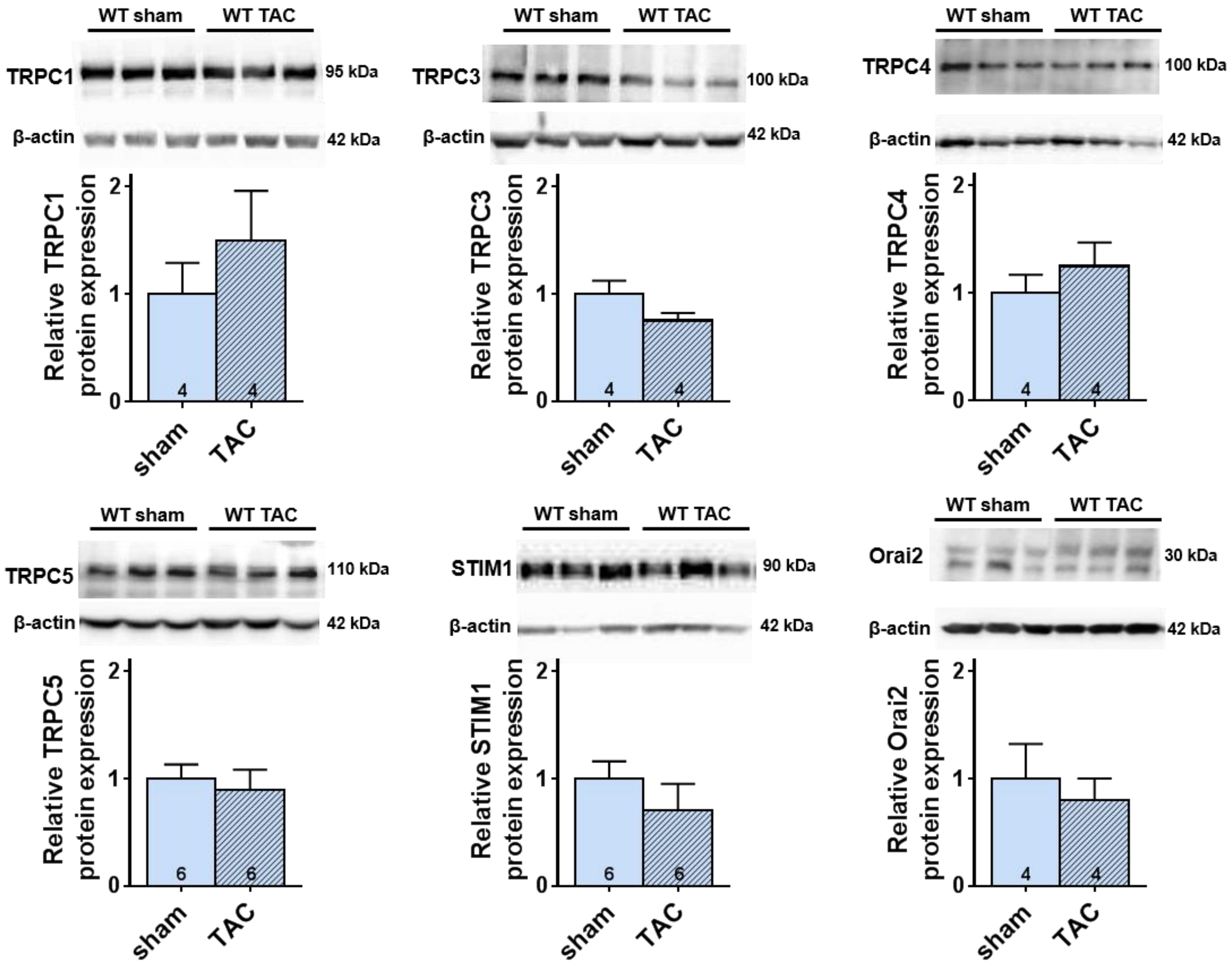

Supplemental Figure 4: TAC does not induce changes in TRPC1, -C3, -C4, -C5, STIM1 and Orai2 expression. Top panels: representative western-blot of TRPC1, -C3, -C4, -C5, STIM1 and Orai2 in ventricle tissue from WT sham or TAC mice after 5 weeks. Bottom panels: relative TRPCs, STIM1 and Orai2 protein expression was evaluated by western-blot. Protein levels were normalized by β-actin and expressed as fold change of that determined in sham mice. N=4-6 animals.

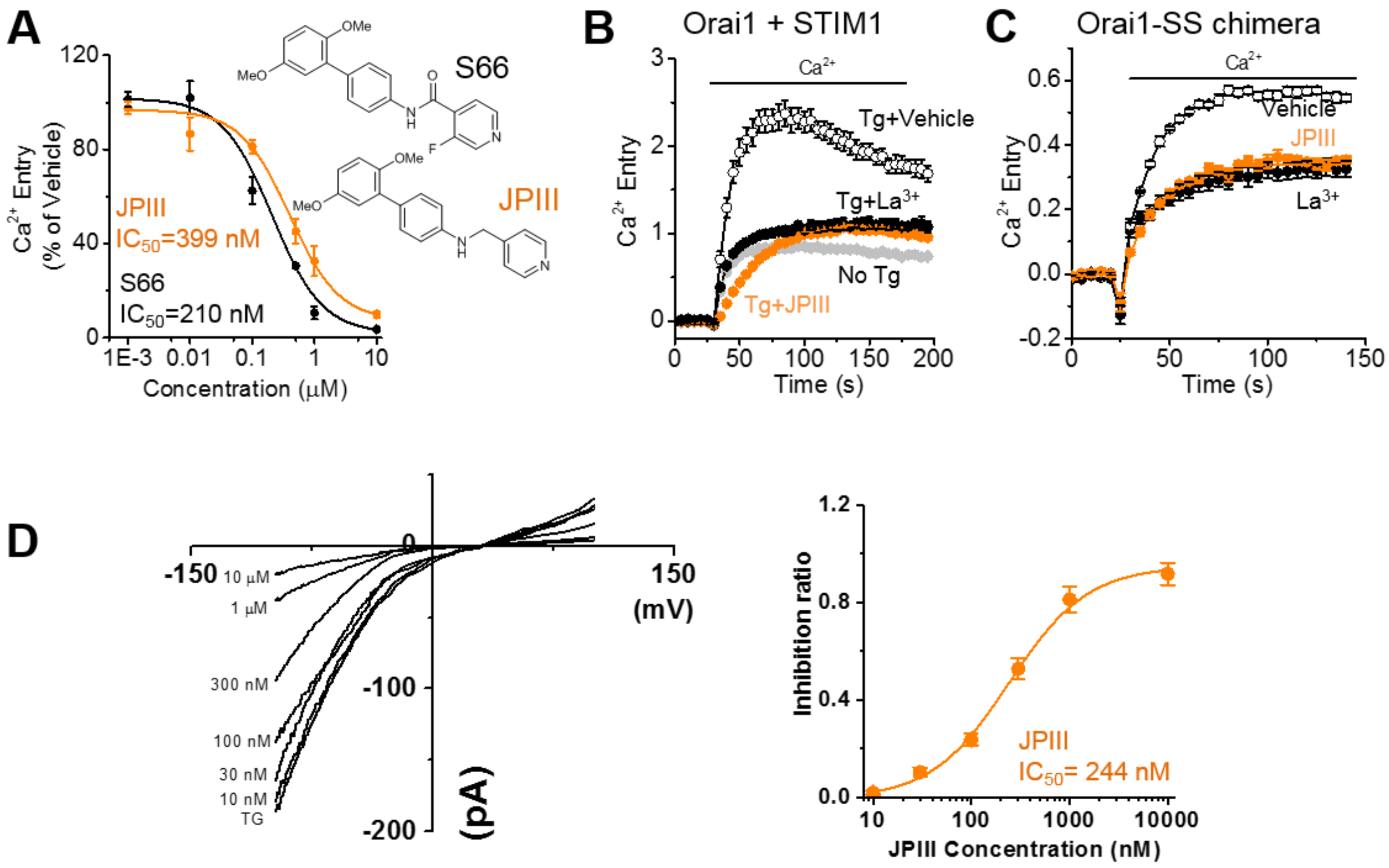

Supplemental Figure 5: The novel S66 analogue, JPIII. **A**, Chemical structure and dose-response for Orai1 inhibition in WT HEK293 cells (JPIII (orange) compared to S66 (black)) following 30 min pre-treatment; the fitted curves are Hill equations for IC_{50} determination. SOCE was stimulated by thapsigargin (Tg) store depletion followed by 0.3 mM Ca^{2+} add back as indicated by the solid line (72 wells in 3 independent experiments). **B-C**, Example FlexStation traces from HEK293 cells transiently transfected with Orai1 and STIM1 or with the constitutively active Orai1-SS chimera. SOCE was stimulated as per (**A**) with JPIII (at 10 µM) compared to 10 µM La^{3+} and vehicle as control (24 wells in 1 experiment). **D**, Orai1 currents were significantly inhibited by JPIII. Left panel: example traces showing that JPIII inhibits Orai1 currents induced by 1 µM Tg. Right panel: dose-response curve shows that the IC_{50} for JPIII inhibitory effect on Orai1 is 244 ± 39 nM (n=6 investigated cells).

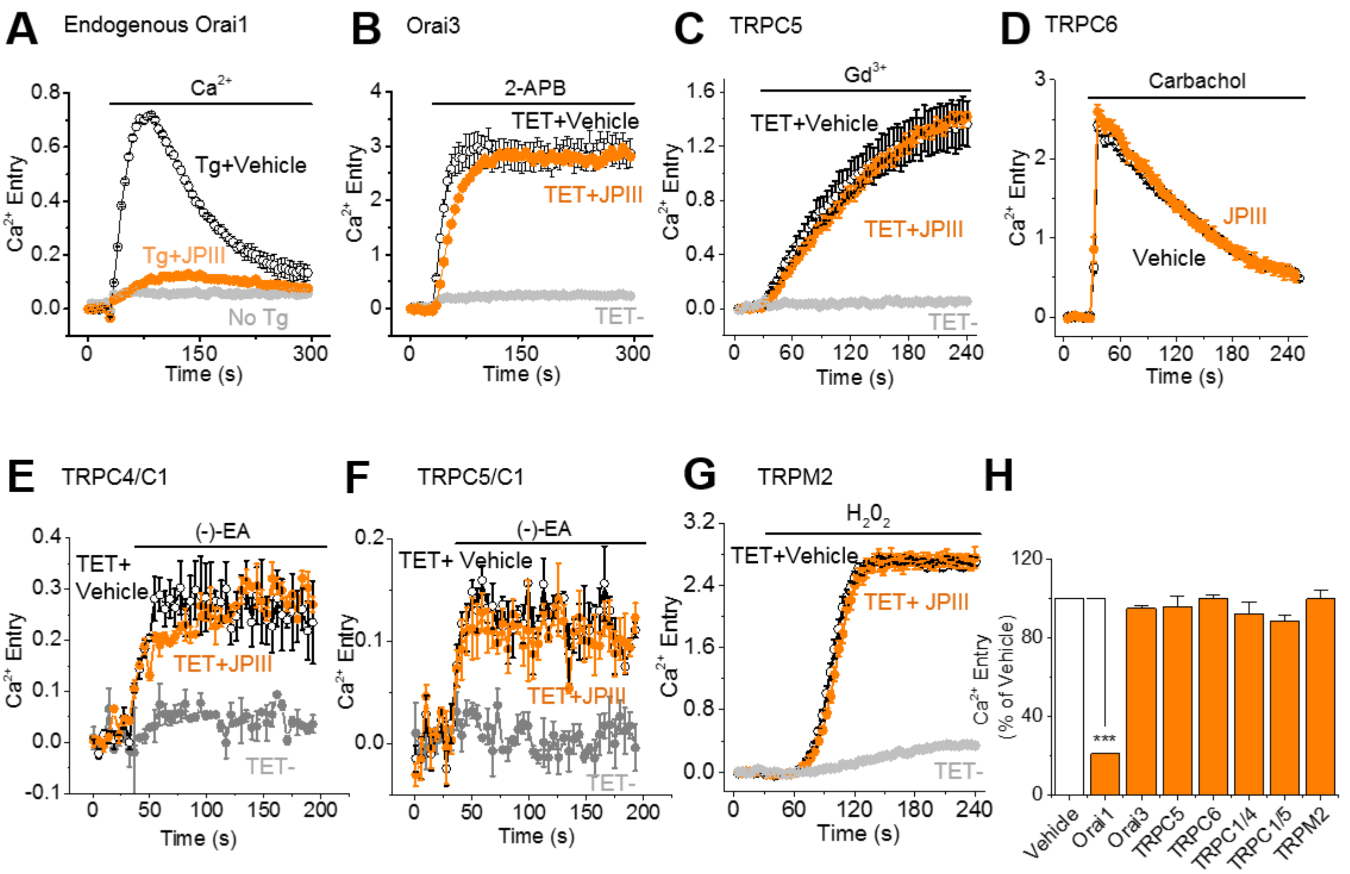

Supplemental Figure 6: Tests of JPIII against multiple types of Ca^{2+}-permeable channels. **A-G**, Intracellular Ca^{2+} recordings are displayed: each panel shows representative mean data from multiple wells in a 96-well plate (24 wells in 1 experiment). Except in the case of recordings from endogenous Orai1 channels in HEK293 cells (**A**), HEK293 cells were stably over-expressing exogenous mouse TRPC6 or stably over-expressing tetracycline-regulated human Orai3, TRPC5, TRPC4-TRPC1 concatemer, TRPC5-TRPC1 concatemer, or TRPM2 (**B-G**). In each case, cells were pre-treated for 30 min with vehicle or JPIII and then maintained in vehicle (black hollow symbols) or 10 µM JPIII (orange). Extracellular Ca^{2+} was continuously present except for the endogenous Orai1 recordings when it was added back to Ca^{2+}-free medium at 0.3 mM after pre-treatment with 1 µM Tg, which depleted intracellular Ca^{2+} stores. Grey symbols show recordings in the absence of Tg (No Tg) or absence of tetracycline induction (TET-). Orai3 channels (**B**) were activated by 75 µM 2-aminoethoxydiphenyl borate (2-APB). TRPC5 channels (**C**) were activated by 20 µM gadolinium (Gd^{3+}). TRPC6 channels (**D**) were activated by 100 µM carbachol, an agonist at endogenous muscarinic receptors of the HEK293 cells (this response includes an initial component of Ca^{2+}-release from intracellular stores, which was not TRPC6-mediated). TRPC4-TRPC1 (**E**) and TRPC5-TRPC1 (**F**) channels were activated by the plant chemical (-)-Englerin A ((-)-EA) at 100 nM. TRPM2 channels (**G**) were stimulated by 1 mM H_2O_2. (**H**) Represents mean data for the types of experiment shown in **A-G** for three independent experiments. Ca^{2+} entry through the various channels is compared after treatment with vehicle or JPIII. ****P*<0.001 *vs.* Vehicle. Statistical significance was evaluated using one-way ANOVA followed by post-hoc Fisher LSD test for multiple comparisons.

Characteristic	Metric	Mean
**Aqueous Solubility†**	Precipitation mean (at pH 7.4) after 2h	36 μM
**Plasma Stability‡** ***Mouse***	Proportion remaining after 2h	97 %
**Plasma Stability‡** ***Human***	Proportion remaining after 2h	100 %
**Plasma Protein Binding†** ***Mouse***	Fraction unbound (fu) in 10% murine plasma	7.9 %
**Metabolic Stability†** ***Mouse***	Intrinsic clearance (CL_{int}) and half life ($t_{1/2}$) in murine liver microsomes	CL_{int}: 554 μL/min/10^6cells $t_{1/2}$: 2.5 min
**Metabolic Stability†** ***Human***	Intrinsic clearance (CL_{int}) and half life ($t_{1/2}$) in human liver microsomes	CL_{int}: 74.6 μL/min/10^6cells $t_{1/2}$: 18.6 min
**Hepatic Stability†** ***Mouse***	Instrinsic clearance (CL_{int}) and half life ($t_{1/2}$) in murine hepatocytes	CL_{int}: 105 μL/min/10^6cells $t_{1/2}$: 13.2 min
**Cell Permeability†** ***Human***	Uni-directional Caco-2 permeability coefficient (P_{app}) after 2h	16.4 x10^{-6} cm/s

Supplemental Figure 7: Pharmacokinetic properties of JPIII *in vitro*. JPIII was assessed for pharmacokinetic characteristics as indicated in the table. For each characteristic, the species is specified (where relevant) in italics in the first column and a brief description of the metric used for quantification along with the results. These experiments were outsourced to Commercial Research Organisations as indicated in the table: † denotes experiments were performed at Cyprotex (Macceslfield, UK), ‡ denotes experiments were performed at Peakdale (High Peak, UK).

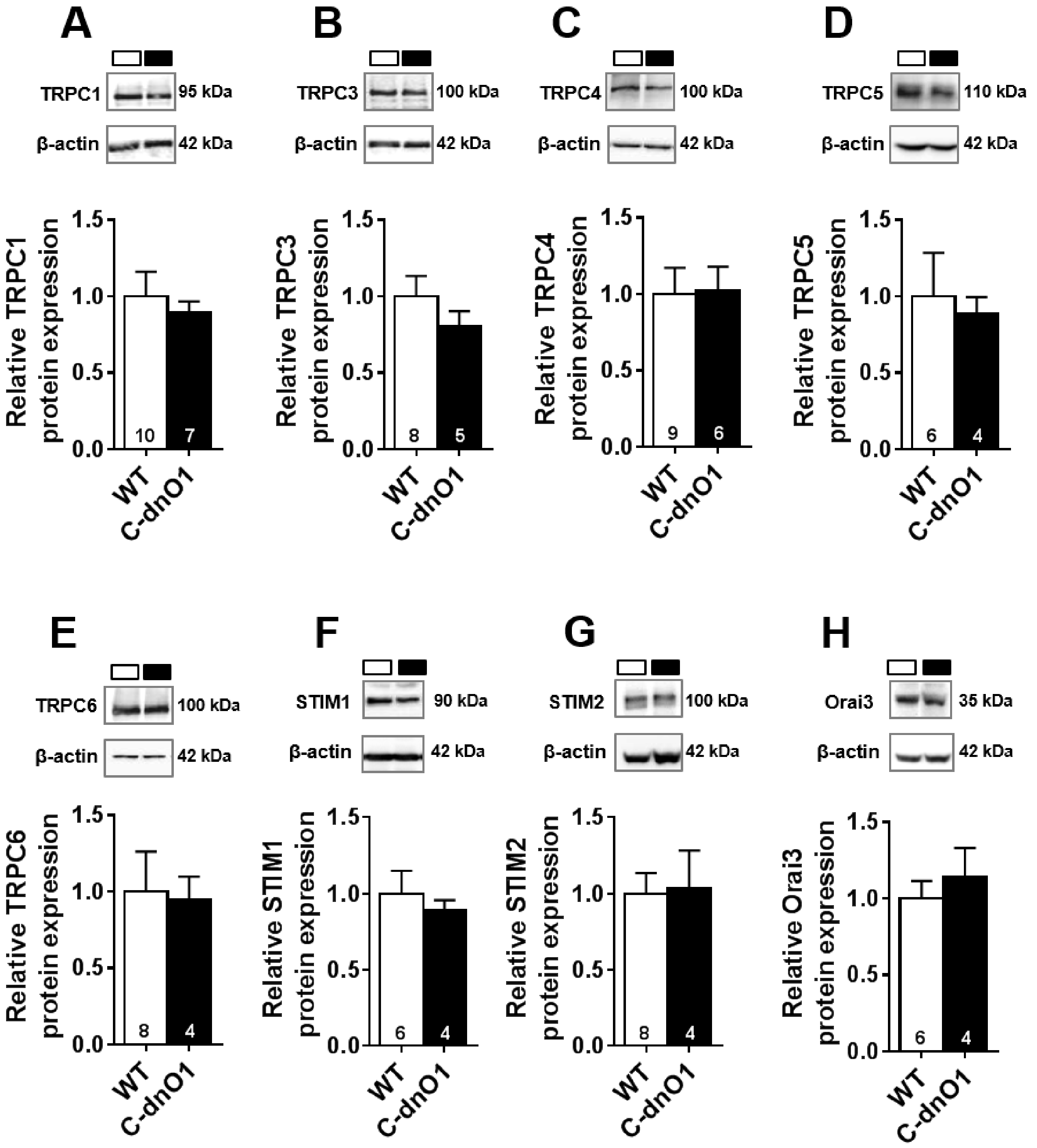

Supplemental Figure 8: C-dnO1 mice do not show any compensation by other SOCs components. **A-H**, Relative protein expression of TRPC1 (**A**), TRPC3 (**B**), TRPC4 (**C**), TRPC5 (**D**), TRPC6 (**E**), STIM1 (**F**), STIM2 (**G**) and Orai3 (**H**) was evaluated by western-blot. Top panels: representative western-blot of SOCs components in ventricle tissue from WT and C-dnO1 mice. Bottom panels: protein levels were normalized by β-actin and expressed as fold change of that determined in WT mice. N=4-10 animals.

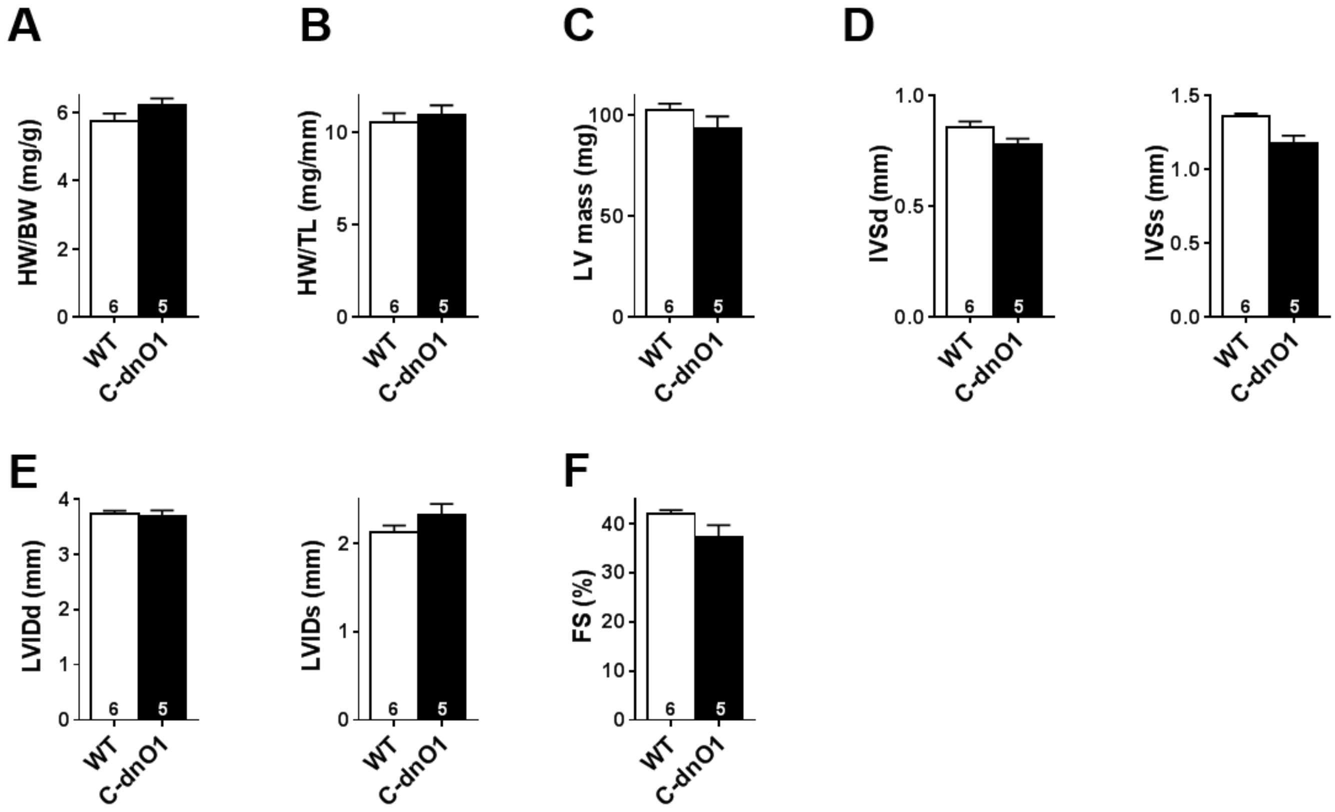

Supplemental Figure 9: C-dnO1 mice display preserved cardiac function at 6 months of age. **A-B**, HW/BW ratio (**A**) and HW/TL ratio (**B**) measured post-mortem in WT and C-dnO1 mice at 6 months of age. **C-E**, LV parameters obtained from analysis of echocardiograms: LV mass (**C**), interventricular septal thickness at end diastole and systole (IVSd and IVSs) (**D**), LV internal diameter at end diastole and systole (LVIDd and LVIDs) (**E**) and cardiac FS % (**F**) in WT and C-dnO1 mice at 6 months of age. N=5-6 animals.

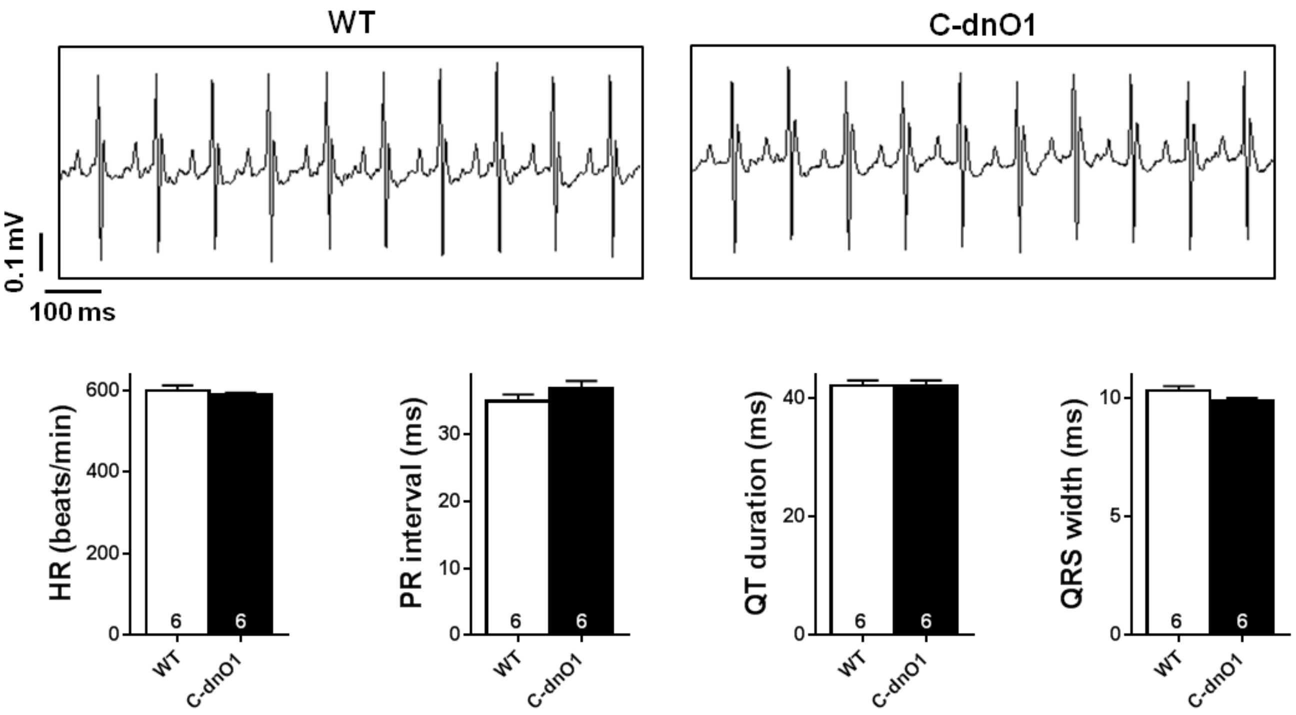

Supplemental Figure 10: C-dnO1 mice display preserved cardiac electrical function. Representative electrocardiograms (ECG) recordings of WT and C-dnO1 mice under basal condition and functional parameters showing the analyzed P, QRS and T components. N=6 animals. Statistical significance was evaluated using Student's t-test (reported P values).

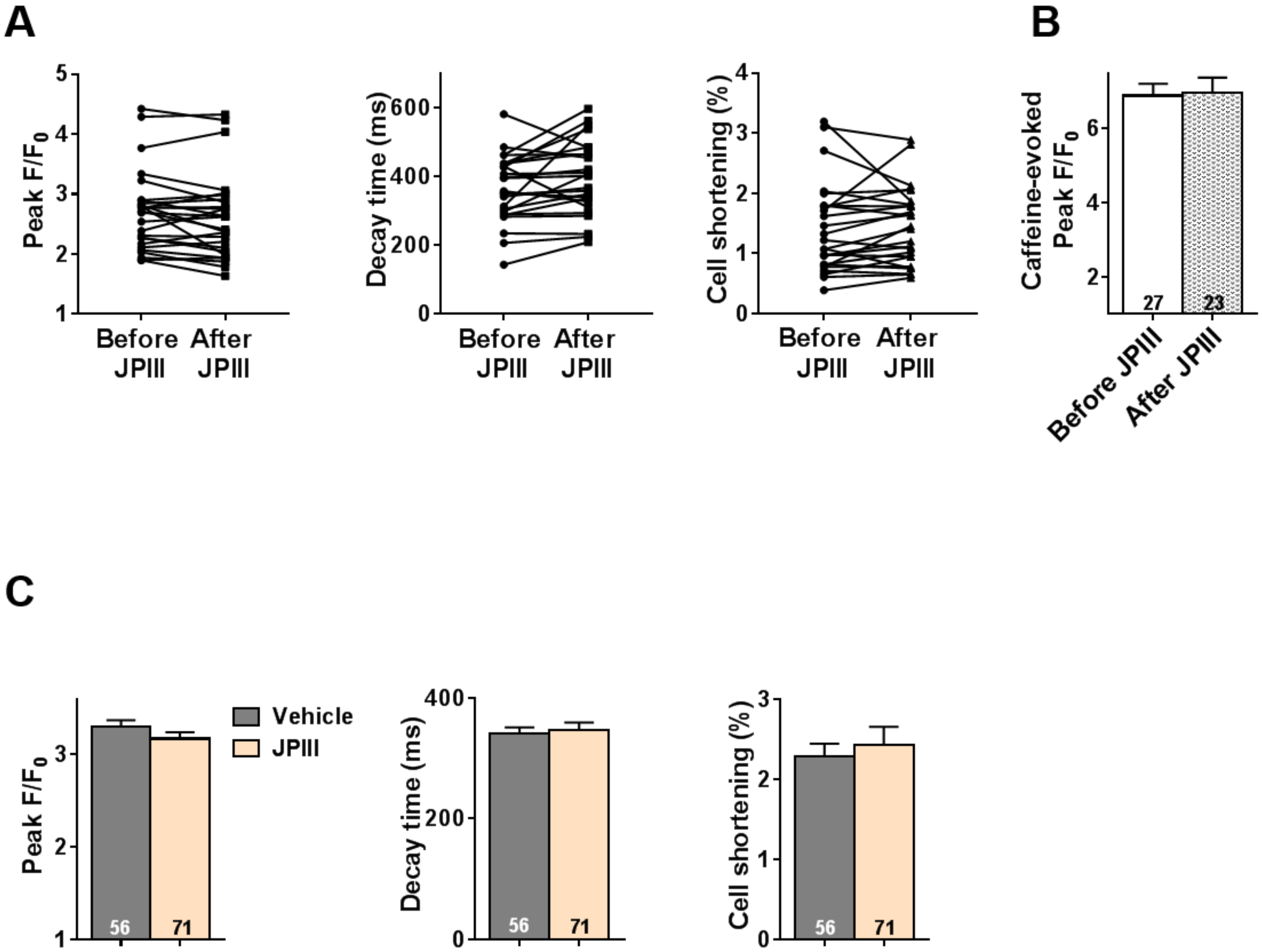

Supplemental Figure 11: Acute or chronic JPIII treatment do not affect Ca^{2+} cycling under basal conditions. **A**, Average amplitude of $[Ca^{2+}]_i$ transients (Peak F/F_0), average of $[Ca^{2+}]_i$ transients decay time constant (ms) and average % of cell shortening before and after acute JPIII application at 5 µM in cardiomyocytes from WT mice field-stimulated at 1 Hz. **B**, Average of amplitude of the caffeine-evoked SR Ca^{2+} load (Peak F/F_0) recorded in cardiomyocytes treated or not with JPIII from WT mice. N=3 animals. n=23-27 investigated cells. **C**, Average amplitude of $[Ca^{2+}]_i$ transients (Peak F/F_0), average of $[Ca^{2+}]_i$ transients decay time constant (ms) and average % of cell shortening in cardiomyocytes from Vehicle or JPIII-treated WT mice for 3 weeks. N=4 animals. n=56-71 investigated cells.

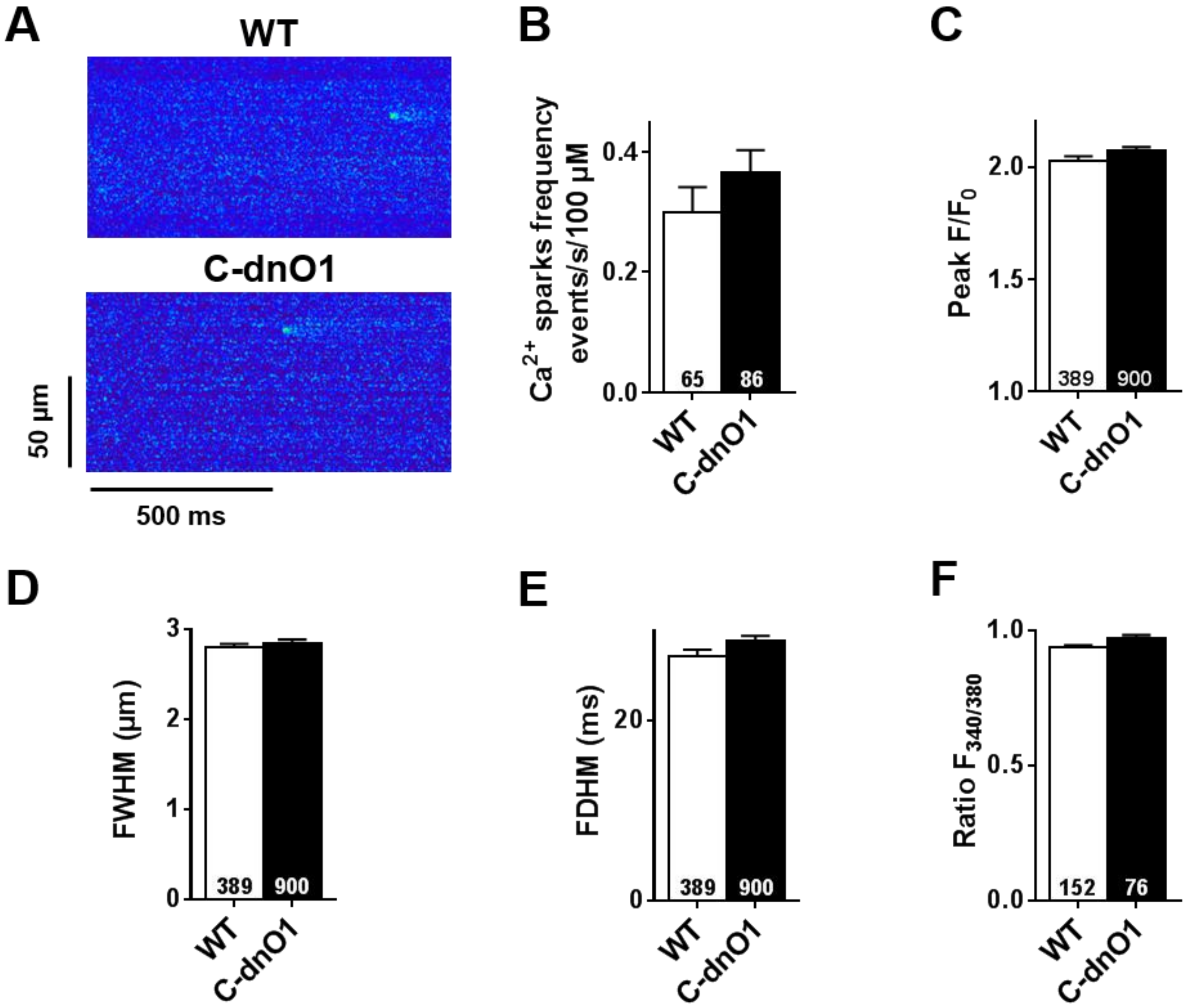

Supplemental Figure 12: Intrinsic RyR cluster activity and structural organization are preserved in C-dnO1 mice. **A**, Representative line-scan images of Ca^{2+} sparks in cardiomyocytes, under rest condition, from WT or C-dnO1 mice. **B**, Ca^{2+} sparks frequency (number of sparks/s/100 µM) recorded from 65 cells from WT mice and 86 cells from C-dnO1 mice. **C**, Ca^{2+} sparks amplitude (measured as peak F/F_0). **D**, Width (FWHM) at 50 % of maximum amplitude. **E**, Duration (FDHM) at 50 % of maximum amplitude. N=3-4 animals. n=389-900 analyzed Ca^{2+} sparks. **F**, Diastolic Ca^{2+} levels determined by Fura-2 (ratio 340/380). N=3-4 animals. n=76-152 investigated cells.

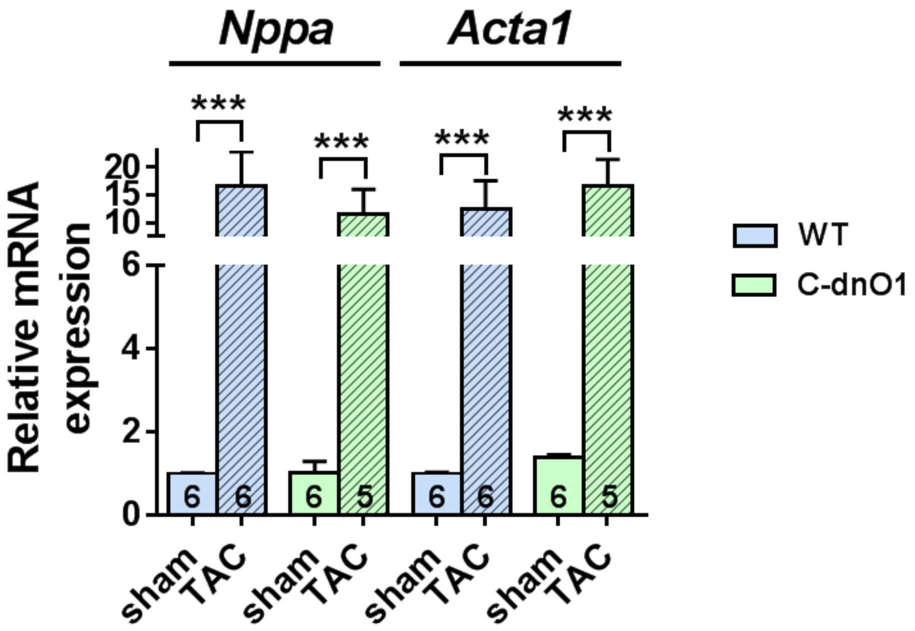

Supplemental Figure 13: Similar induction of pro-hypertrophic markers in WT and C-dnO1 mice after TAC. Atrial natriuretic factor (*Nppa*) and skeletal α-actin (*Acta1*) mRNA expression was determined by RT-qPCR in ventricle tissue from WT and C-dnO1 sham or TAC mice after 5 weeks. mRNA levels were normalized to housekeeping genes and expressed as fold change of that determined in sham WT mice. N=5-6 animals. *** P<0.001 *vs.* respective sham mice. Statistical significance was evaluated using two-way ANOVA followed by post-hoc Fisher LSD test for multiple comparisons.

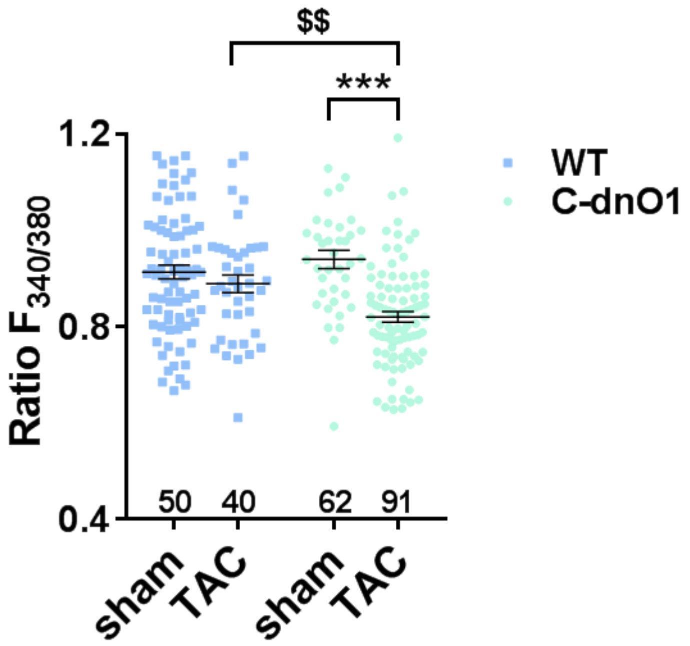

Supplemental Figure 14: Diastolic Ca^{2+} levels determined by Fura-2 (ratio 340/380) in WT and C-dnO1 mice after TAC. N=3-4 animals. n=40-91 investigated cells. ****P*<0.001 *vs.* C-dnO1 sham mice. $^{\$\$}$*P*<0.01 *vs.* WT TAC mice. Statistical significance was evaluated using two-way ANOVA followed by post-hoc Fisher LSD test for multiple comparisons.

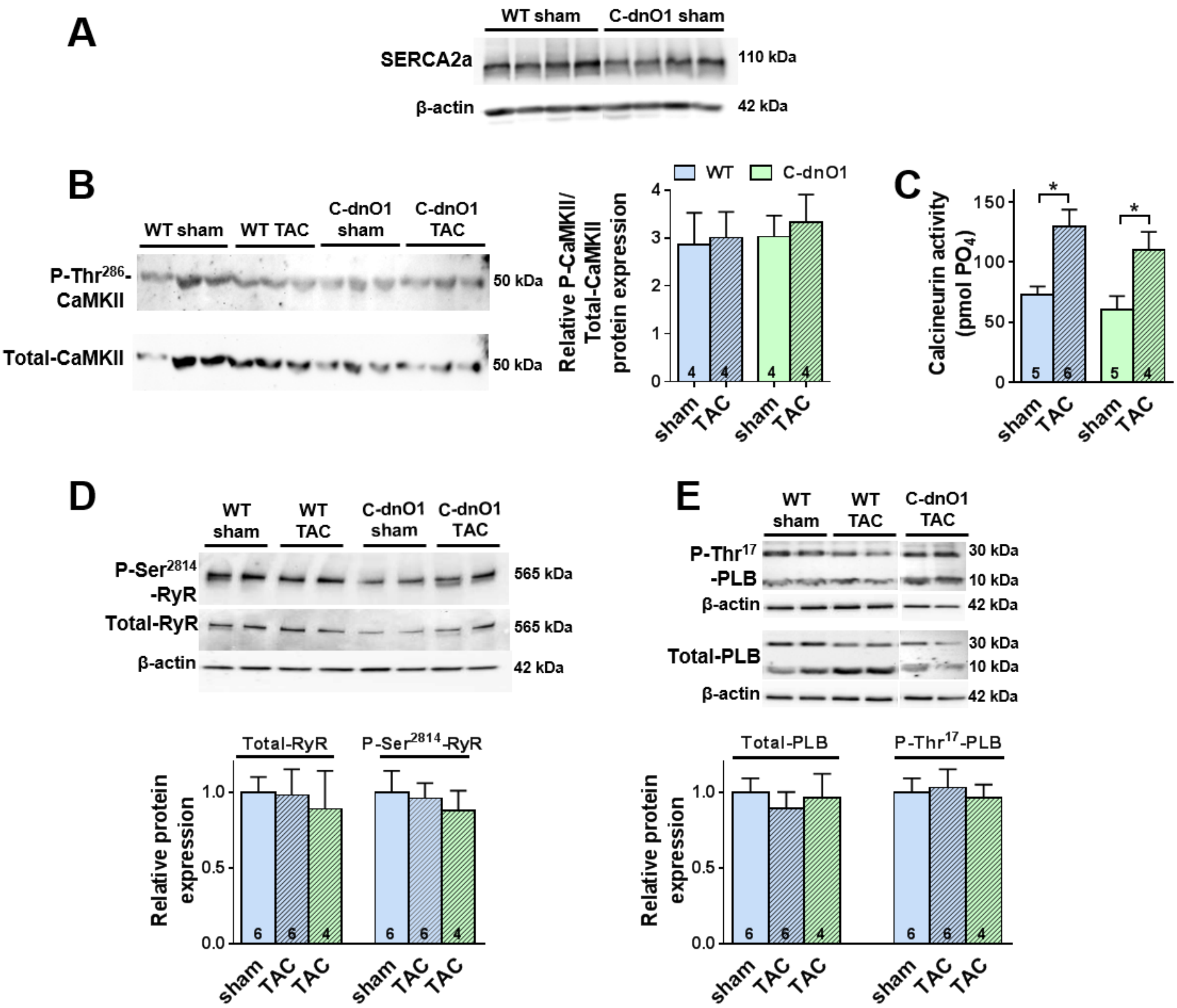

Supplemental Figure 15: CaMKII, CaN activities, RyR and PLB phosphorylation states are not changed after TAC. **A**, Representative western-blot of SERCA2a in ventricle tissue from sham WT and C-dnO1 mice. **B**, Representative western-blot and quantification of P-thr^{286}-CaMKII and Total-CaMKII in ventricle tissue from sham and TAC mice. Protein levels were normalized by β-actin and expressed as fold change of that determined in sham mice. N=4 animals. **C**, Cellular calcineurin phosphatase activity (in pmol of phosphate released) in ventricle tissue from sham and TAC mice. N=4-6 animals. $^{*}P<0.05$ *vs.* respective sham mice. **D-E**, Representative western-blot and quantification of P-ser^{2814}-RyR2 and Total-RyR2 (**D**) and of P-thr^{17}-PLB and Total-PLB expression (**E**) in ventricle tissue from sham and 5 weeks post-TAC mice. Protein levels were normalized by β-actin and expressed as fold change of that determined in sham mice. N=4-6 animals. Statistical significance was evaluated using two-way ANOVA followed by post-hoc Fisher LSD test for multiple comparisons.

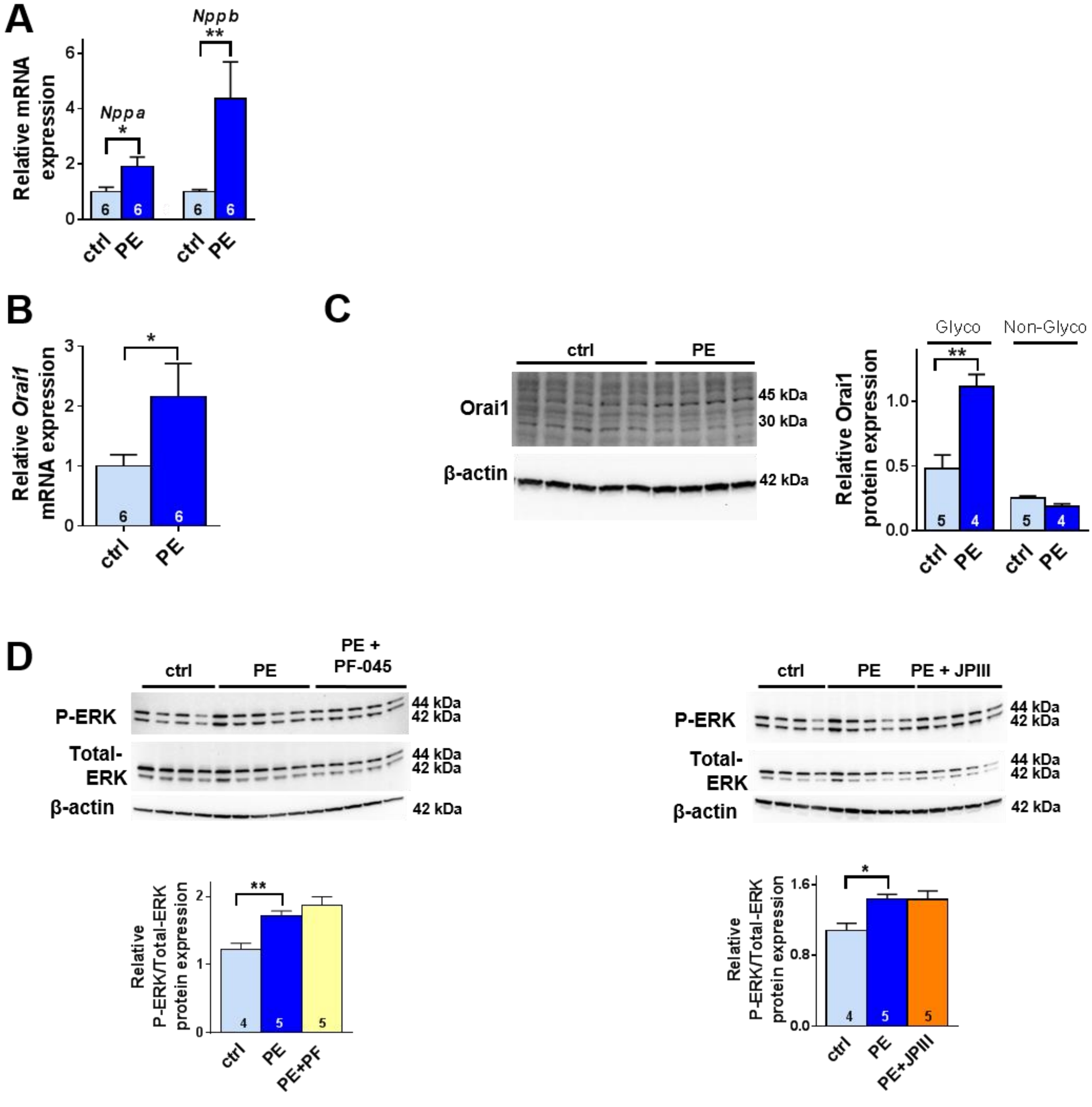

Supplemental Figure 16: 48h PE induces increased Orai1 expression and ERK1/2 activity in NRVMs. **A**-**B,** Relative *Nppa and Nppb* (natriuretic peptide B) (**A**) and *Orai1* (**B**) mRNA expression was determined by RT-qPCR in ctrl or 100 µM phenylephrine (PE)-treated neonatal rat ventricular cardiomyocytes (NRVMs) for 48 h. mRNA levels were normalized to housekeeping genes and expressed as fold change of that determined in ctrl NRVMs. **C**, Representative western-blot of Orai1 (left panel) and relative Orai1 protein expression (right panel) was evaluated by western-blot in ctrl or PE-treated NRVMs. **D**, Representative western-blots and quantifications of P-ERK1/2 and Total-ERK1/2 in ctrl or PE-treated NRVMs in presence of PF-04520440 or JPIII. Phosphorylation levels were normalized by Total-ERK1/2 expression and expressed as fold change of that determined in ctrl NRVMs.

N=4-6 primary cultures. *$P<0.05$, **$P<0.01$ *vs.* ctrl NRVMs. Statistical significance was evaluated using Student's *t*-test (**A-C**) and one-way ANOVA followed by post-hoc Fisher LSD test for multiple comparisons in **D**.

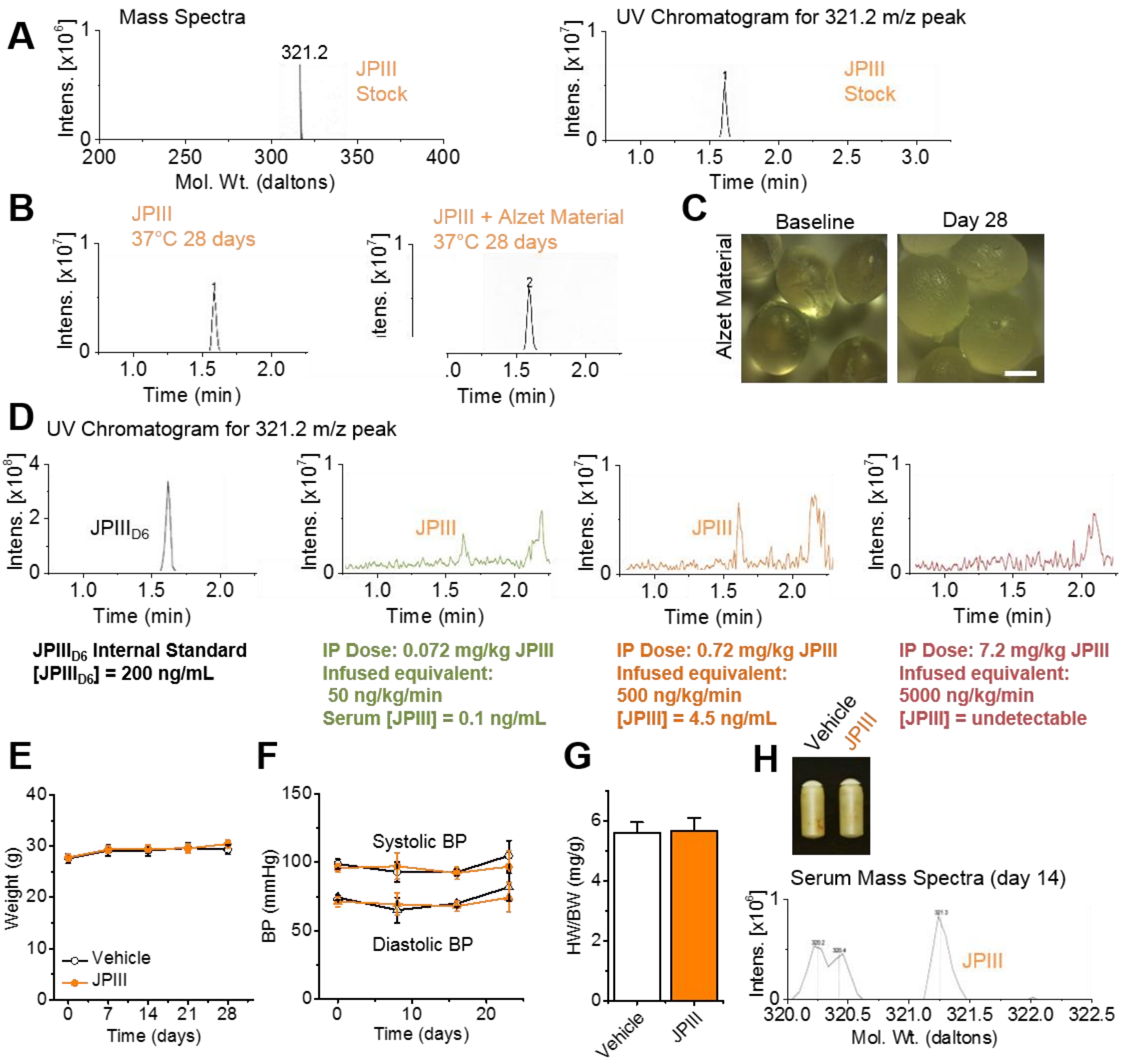

Supplemental Figure 17: JPIII *in vivo* dose finding. **A-B**, Compatibility of JPIII with the Alzet osmotic mini-pumps. For *in vivo* delivery, JPIII was dissolved in dimethylsulfoxide and delivered at 500 ng/kg/min. **A**, Left panel: representative mass spectra obtained by LC-MS for the fresh JPIII stock solution, with a single peak at 321.2m/z. Right panel: UV chromatogram LC-MS traces for the extracted ion at 321.2m/z of fresh JPIII stock solution. **B**, JPIII stock solution after incubation at 37°C for 28 days (left panel) and after incubation at 37°C for 28 days in conjunction with the Alzet pump osmotic material (+Alzet Material, right panel). **C**, Bright field images of the polymer balls before and after incubation with the JPIII solvent for 28 days. Scale bar, 5 mm. **D**, Example UV chromatogram LC-MS for the extracted ion peak at 321.2m/z (which corresponds to JPIII) for the internal standard $JPIII_{D6}$ and from serum extracted 15 min after intra-peritoneal administration of 0.072 mg/kg (green trace), 0.72 mg/kg (orange trace) and 7.2 mg/kg (red trace). In each case, the sample was spiked with the $JPIII_{D6}$ internal standard to allow quantification of concentration. **E-F**, Lack

of effect of JPIII on body weight or basic physiological cardiovascular parameters. JPIII (500 ng/kg/min) or vehicle only were delivered to C57BL6/J mice by subcutaneously implanted osmotic mini-pump for 28 days. **E**, Mean data for weight of animals from the two groups during infusion (vehicle is the open symbols, JPIII the orange symbols). N=4-5 animals. **F**, Mean data from weekly mean systolic (circles) and diastolic (triangles) blood pressure during the infusion period by tail cuff plethysmography (vehicle is the open symbols, JPIII the orange symbols). N=4-5 animals. **G**, Quantification of mean HW/BW ratio. N=4-5 animals. **H**, LC-MS ion peak for JPIII from saphenous vein serum on day 14 of infusion. Inset panel: images of osmotic mini-pumps harvested from animals after 28 days infusion of vehicle or JPIII.

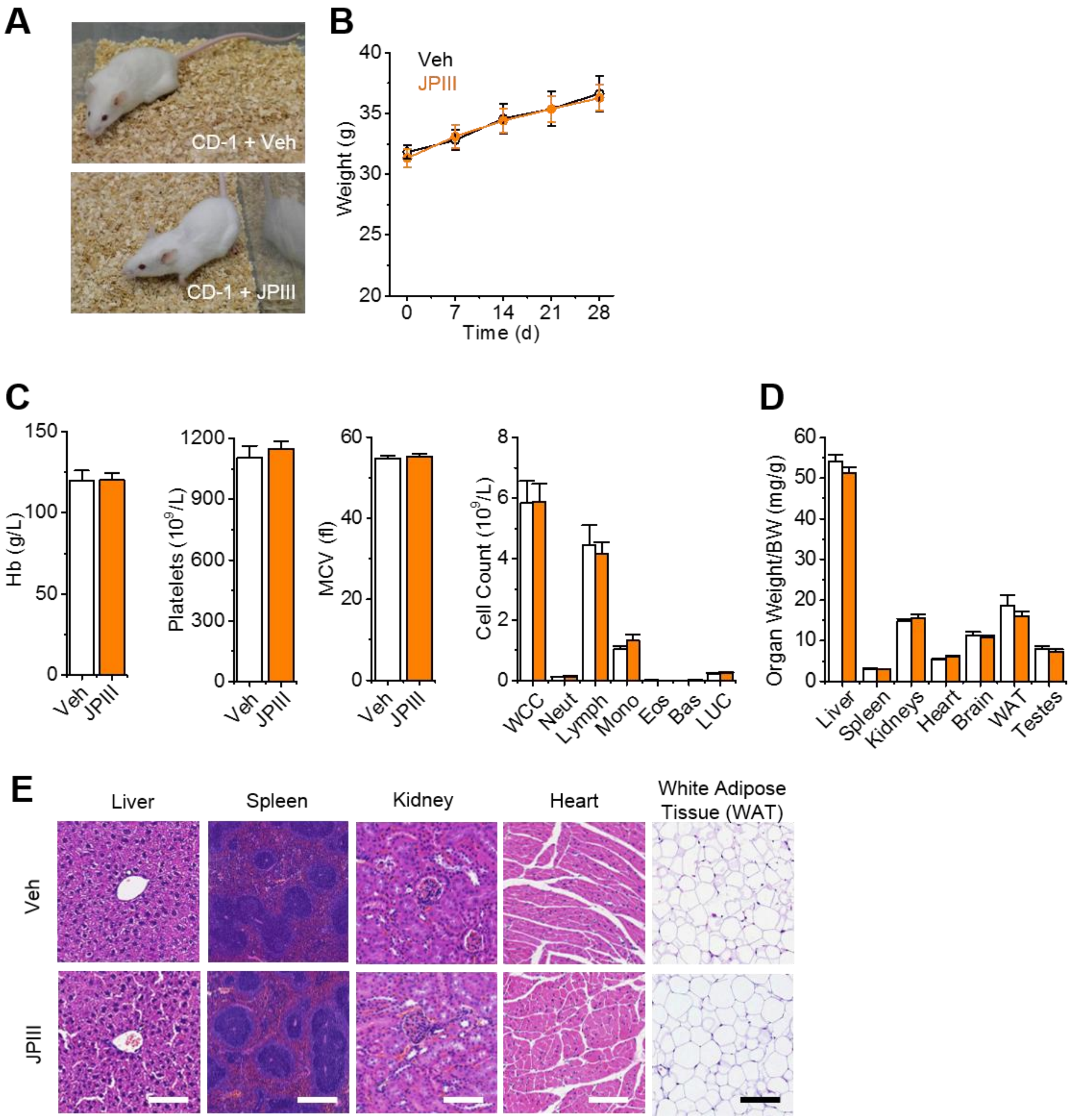

Supplemental Figure 18: JPIII does not exhibit major toxic flags *in vivo*. **A**, Example images of the CD-1 mice from the JPIII and vehicle infused group at the end of the 28 day infusion period. **B**, Mean data for animal weights throughout the study. **C**, Haematological assessment; Hb = haemoglobin; MCV = mean cell volume; WCC = total white cell count; for differential white cell count, data are presented for: Neut = neutrophils; Lymph = lymphocytes; Mono = monocytes; Eos = eosinophils; Bas = basophils; LUC = large unstained cells. **D**, Mean data for the organ to body weight ratios. WAT = white adipose tissue. **E**, Representative H&E stained organ sections for the data in (**D**), scale bars are 100 microns except for spleen where scale bar is 400 microns. For all panels N=12 animals. No significant differences were observed between groups for any parameters.

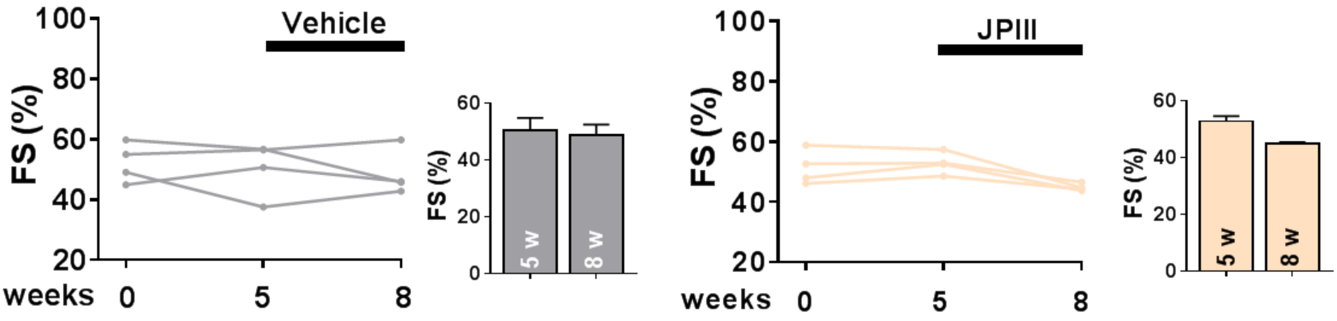

Supplemental Figure 19: Systolic function in sham-operated mice treated or not with JPIII. Left panels: time-dependent effect of vehicle and JPIII on LV FS (%) obtained from analysis of echocardiograms in sham mice. Right panels: average of FS (%) in sham-operated mice. N=4 animals.

Supplemental References

1. Ainscough JF, John RM and Barton SC. Production of YAC transgenic mice by pronuclear injection. *Methods Mol Biol*. 2001;181:55-65.

2. Brooke DA, Orsi NM, Ainscough JF, Holwell SE, Markham AF and Coletta PL. Human menopausal and pregnant mare serum gonadotrophins in murine superovulation regimens for transgenic applications. *Theriogenology*. 2007;67:1409-1413.

3. Redfern CH, Coward P, Degtyarev MY, Lee EK, Kwa AT, Hennighausen L, Bujard H, Fishman GI and Conklin BR. Conditional expression and signaling of a specifically designed Gi-coupled receptor in transgenic mice. *Nat Biotechnol*. 1999;17:165-169.

4. Leroy J, Richter W, Mika D, Castro LR, Abi-Gerges A, Xie M, Scheitrum C, Lefebvre F, Schittl J, Mateo P et al. Phosphodiesterase 4B in the cardiac L-type Ca^{2+} channel complex regulates Ca^{2+} current and protects against ventricular arrhythmias in mice. *J Clin Invest*. 2011;121:2651-2661.

5. Sabourin J, Bartoli F, Antigny F, Gomez AM and Benitah JP. Transient Receptor Potential Canonical (TRPC)/Orai1-dependent Store-operated Ca^{2+} Channels: NEW TARGETS OF ALDOSTERONE IN CARDIOMYOCYTES. *J Biol Chem*. 2016;291:13394-13409.

6. McHugh D, Flemming R, Xu SZ, Perraud AL and Beech DJ. Critical intracellular Ca^{2+} dependence of transient receptor potential melastatin 2 (TRPM2) cation channel activation. *J Biol Chem*. 2003;278:11002-11006.

7. Xu SZ, Zeng F, Boulay G, Grimm C, Harteneck C and Beech DJ. Block of TRPC5 channels by 2-aminoethoxydiphenyl borate: a differential, extracellular and voltage-dependent effect. *Br J Pharmacol*. 2005;145:405-414.

8. Zeng F, Xu SZ, Jackson PK, McHugh D, Kumar B, Fountain SJ and Beech DJ. Human TRPC5 channel activated by a multiplicity of signals in a single cell. *J Physiol*. 2004;559:739-750.

9. Akbulut Y, Gaunt HJ, Muraki K, Ludlow MJ, Amer MS, Bruns A, Vasudev NS, Radtke L, Willot M, Hahn S et al. (-)-Englerin A is a potent and selective activator of TRPC4 and TRPC5 calcium channels. *Angew Chem Int Ed Engl*. 2015;54:3787-3791.

10. Naylor J, Minard A, Gaunt HJ, Amer MS, Wilson LA, Migliore M, Cheung SY, Rubaiy HN, Blythe NM, Musialowski KE et al. Natural and synthetic flavonoid modulation of TRPC5 channels. *Br J Pharmacol*. 2016;173:562-574.

11. Li Z, Liu L, Deng Y, Ji W, Du W, Xu P, Chen L and Xu T. Graded activation of CRAC channel by binding of different numbers of STIM1 to Orai1 subunits. *Cell Res*. 2011;21:305-315.

12. Somasundaram A, Shum AK, McBride HJ, Kessler JA, Feske S, Miller RJ and Prakriya M. Store-operated CRAC channels regulate gene expression and proliferation in neural progenitor cells. *J Neurosci*. 2014;34:9107-123.

13. Dominguez-Rodriguez A, Ruiz-Hurtado G, Sabourin J, Gomez AM, Alvarez JL and Benitah JP. Proarrhythmic effect of sustained EPAC activation on TRPC3/4 in rat ventricular cardiomyocytes. *J Mol Cell Cardiol*. 2015;87:74-78.

14. Lambert V, Gouadon E, Capderou A, Le Bret E, Ly M, Dinanian S, Renaud JF, Puceat M and Rucker-Martin C. Right ventricular failure secondary to chronic overload in

congenital heart diseases: benefits of cell therapy using human embryonic stem cell-derived cardiac progenitors. *J Thorac Cardiovasc Surg*. 2015;149:708-715 e1.

15. Sabourin J, Boet A, Rucker-Martin C, Lambert M, Gomez AM, Benitah JP, Perros F, Humbert M and Antigny F. Ca^{2+} handling remodeling and STIM1L/Orai1/TRPC1/TRPC4 upregulation in monocrotaline-induced right ventricular hypertrophy. *J Mol Cell Cardiol*. 2018;118:208-224.

16. Ouvrard-Pascaud A, Sainte-Marie Y, Benitah JP, Perrier R, Soukaseum C, Nguyen Dinh Cat A, Royer A, Le Quang K, Charpentier F, Demolombe S et al. Conditional mineralocorticoid receptor expression in the heart leads to life-threatening arrhythmias. *Circulation*. 2005;111:3025-3033.

17. Derumeaux G, Mulder P, Richard V, Chagraoui A, Nafeh C, Bauer F, Henry JP and Thuillez C. Tissue Doppler imaging differentiates physiological from pathological pressure-overload left ventricular hypertrophy in rats. *Circulation*. 2002;105:1602-1608.

18. Weingand K, Bloom J, Carakostas M, Hall R, Helfrich M, Latimer K, Levine B, Neptun D, Rebar A, Stitzel K and et al. Clinical pathology testing recommendations for nonclinical toxicity and safety studies. AACC-DACC/ASVCP Joint Task Force. *Toxicol Pathol*. 1992;20:539-543.

19. Weingand K, Brown G, Hall R, Davies D, Gossett K, Neptun D, Waner T, Matsuzawa T, Salemink P, Froelke W et al. Harmonization of animal clinical pathology testing in toxicity and safety studies. The Joint Scientific Committee for International Harmonization of Clinical Pathology Testing. *Fundam Appl Toxicol*. 1996;29:198-201.
